# ADAR and hnRNPC deficiency synergize in activating endogenous dsRNA-induced type I IFN responses

**DOI:** 10.1084/jem.20201833

**Published:** 2021-07-23

**Authors:** Anna-Maria Herzner, Zia Khan, Eric L. Van Nostrand, Sara Chan, Trinna Cuellar, Ronald Chen, Ximo Pechuan-Jorge, Laszlo Komuves, Margaret Solon, Zora Modrusan, Benjamin Haley, Gene W. Yeo, Timothy W. Behrens, Matthew L. Albert

**Affiliations:** 1 Department of Cancer Immunology, Genentech, South San Francisco, CA; 2 Department of Human Genetics, Genentech, South San Francisco, CA; 3 Department of Cellular and Molecular Medicine, Stem Cell Program and the Institute for Genomic Medicine, University of California, San Diego, La Jolla, CA; 4 Department of Pathology, Genentech, South San Francisco, CA; 5 Department of Molecular Biology, Genentech, South San Francisco, CA; 6 Department of Microchemistry, Proteomics & Lipidomics and Next Generation Sequencing, Genentech, South San Francisco, CA

## Abstract

Cytosolic double-stranded RNA (dsRNA) initiates type I IFN responses. Endogenous retroelements, notably Alu elements, constitute a source of dsRNA. Adenosine-to-inosine (A-to-I) editing by ADAR induces mismatches in dsRNA and prevents recognition by MDA5 and autoinflammation. To identify additional endogenous dsRNA checkpoints, we conducted a candidate screen in THP-1 monocytes and found that hnRNPC and ADAR deficiency resulted in synergistic induction of MDA5-dependent IFN responses. RNA-seq analysis demonstrated dysregulation of Alu-containing introns in hnRNPC-deficient cells via utilization of unmasked cryptic splice sites, including introns containing ADAR-dependent A-to-I editing clusters. These putative MDA5 ligands showed reduced editing in the absence of ADAR, providing a plausible mechanism for the combined effects of hnRNPC and ADAR. This study contributes to our understanding of the control of repetitive element–induced autoinflammation and suggests that patients with hnRNPC-mutated tumors might maximally benefit from ADAR inhibition-based immunotherapy.

## Teaser

hnRNPC prevents release of inverted-repeat Alu double-stranded RNA into the cytosol by masking cryptic splice sites. Herzner et al. found that deficiency in hnRNPC led to dysregulation of splicing and increased abundance of intronic double-stranded RNA, which together with ADAR deficiency resulted in a synergistic increase in spontaneous MDA5-dependent IFN responses.

## Introduction

Cytosolic double-stranded RNA (dsRNA) is a hallmark of viral infection, serving as a trigger for retinoic acid-inducible gene I (RIG-I)–like receptors and the subsequent induction of an antiviral type I IFN response (Schlee and Hartmann, 2016). Endogenous dsRNA may also engage cytosolic sensors (Schlee and Hartmann, 2016), posing an inflammatory threat to homeostasis and acting as a potential driver of autoinflammatory diseases, including interferonopathies (Davidson et al., 2018). One well-characterized safeguard is the double-stranded RNA-specific adenosine deaminase (ADAR, also known as ADAR1). Notably, patients harboring pathological variants of ADAR suffer from Aicardi-Goutières syndrome, with evidence of high levels of circulating IFNs, IFN-stimulated genes (ISGs), and severe encephalopathy (Fisher and Beal, 2017; Frassinelli et al., 2021). Gene deletion or enzymatic mutants of ADAR in mice lead to embryonic lethality at about embryonic day 9.5 or 11.5, respectively, a phenotype that can be rescued by deficiency in the RIG-I–like helicase melanoma differentiation-associated protein 5 (MDA5; Pestal et al., 2015; Liddicoat et al., 2015). These studies have led to a model whereby ADAR is responsible for editing adenosine-to-inosine (A-to-I) within dsRNA stretches of RNA, rendering it inert to MDA5 sensing (Pestal et al., 2015; Liddicoat et al., 2015). Complementing these genetic studies, ADAR inactivation in tumors has been shown to effectively enhance checkpoint blockade by boosting MDA5-dependent immune responses (Ishizuka et al., 2019). In addition, ADAR inactivation has been suggested to act in a cell-intrinsic manner, regulating tumor cell growth through activation of IFN-induced, dsRNA-activated protein kinase (Gannon et al., 2018; Liu et al., 2019). Indeed, ADAR inhibitors are being considered for use in cancer immunotherapy (Ishizuka et al., 2019). Checkpoint inhibitors have been widely successful but require a high immune infiltration for optimal efficacy (Cogdill et al., 2017). One strategy to overcome this requirement constitutes intratumoral innate immune activation. This can be achieved by engaging cytosolic receptor ligands and has been validated as a means to increase immune cell infiltration in tumors (Iurescia et al., 2018). One inherent problem with this strategy is the narrow therapeutic index, as both tumor and host cells express cytosolic nucleic acid sensors. This is being addressed by targeting agonists to tumors. An alternative strategy might be the identification of factors that are dysregulated in tumor cells, which can be exploited to enhance the activity of endogenous ligands for nucleic acid sensors.

Approximately 90–95% of reported ADAR editing sites in human cells are found in Alu element–derived RNA (Bahn et al., 2015; Franzén et al., 2018; Giacopuzzi et al., 2018; Quinones-Valdez et al., 2019). The Alu retrotransposon has uniquely emerged in primates and has the highest copy number of all transposable elements—estimated to be >10^6^ elements that constitute an estimated 11% of the human genome (Deininger, 2011). The abundance of Alu elements prompted us to hypothesize the presence of additional RNA-binding proteins as being involved in preventing Alu-derived dsRNA recognition and sterile inflammation.

In this study, we identified heterogeneous nuclear ribonucleoprotein C (hnRNPC) as an important splicing regulator working together with ADAR by suppressing cytosolic access of Alu-element–derived dsRNA.

## Results

### Targeting hnRNPC and ADAR by CRISPR/Cas9 synergistically activates MDA5

**Figure S1.**
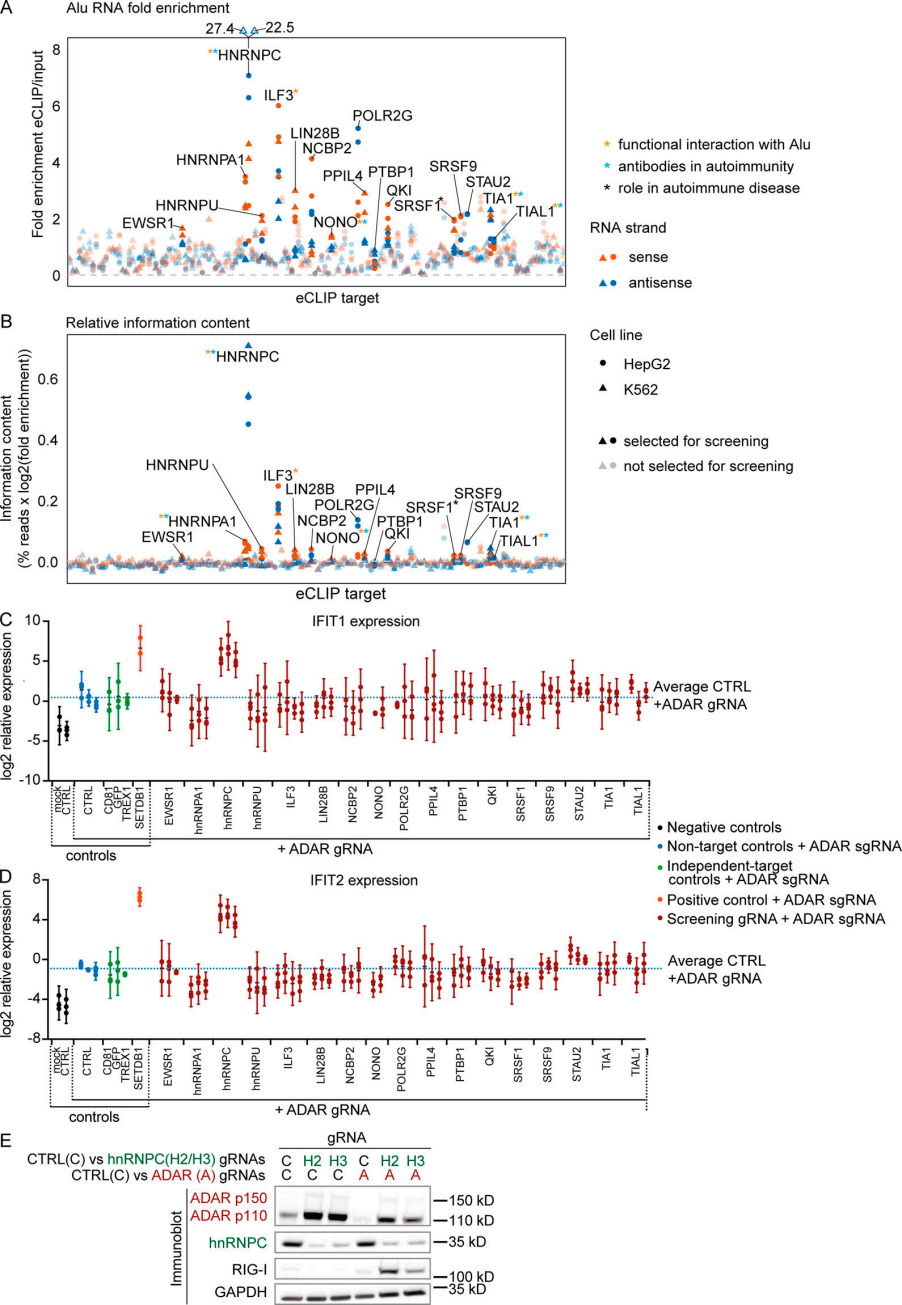
**Selection of screen candidates and IFIT1/2 expression in screen.(A and B)** Fold enrichment (A) and relative information content (B) of Alu-element RNA for 150 eCLIP targets in alphabetical order. The targets selected for the screen are opaque and labeled. Colors indicate RNA strand: red, Alu RNA in sense orientation; dark blue, Alu RNA in antisense orientation. Shapes indicate cell line: circles, HepG2; triangles, K562. Asterisks marking the labels indicate literature showing functional interaction of the target with Alu RNA (orange), antibodies recognizing the target in autoimmunity (light blue), or the target playing a functional role in autoimmune disease (black). References are listed in Table S1. **(C and D)** IFIT1 (C) and IFIT2 (D) expression in THP-1 as in Fig. 1 B. Each set of aligned circles represents unique combinations of RBP targeting + ADAR gRNA (two to four individual gRNAs per RBP). Black, no ADAR targeting; blue, ADAR targeting+nontarget controls; green, ADAR targeting+non–RBP-negative controls; orange, ADAR-targeting + SETDB1 targeting–positive control; red, ADAR targeting+RBP targeting. Dotted blue line indicates average IFIT1 (C) or IFIT2 (D) expression across all ADAR+nontarget gRNA nucleofected samples. Log2 expression from three independent experiments is reported. Bars indicate 95% confidence intervals around mean. Successful CRISPR/Cas9 targeting was not confirmed at this stage. **(E)** Protein detection in THP-1 by Western blot as indicated in Fig. 1 C. One representative out of three experiments. gRNAs: C, Non-target control; H2/H3 hnRNPC gRNA #2 (used in Fig. 2 C); and #3, A, ADAR gRNA. CTRL, nontarget control; sgRNA, single-guide RNA.

To discover previously uncharacterized host factors that prevent dsRNA recognition, we used available enhanced cross-linking and immunoprecipitation (eCLIP) data to identify Alu element RNA-binding proteins (Alu-RBPs; Van Nostrand et al., 2016; Hung et al., 2015). Enrichment at Alu elements was determined by the mapping of eCLIP and input reads to 186 families of RNA (e.g., ribosomal RNAs, transfer RNAs, and noncoding RNAs), including several families of repetitive elements such as Alu or L1 (large interspersed nuclear element 1), separately in sense and antisense orientation (Van Nostrand et al., 2020a; Van Nostrand et al., 2020b). For this analysis, we used fold enrichment in eCLIP over input samples and relative information content (fraction of family-specific eCLIP reads of all eCLIP reads, multiplied by the log-fold enrichment) to select 17 Alu-RBP candidates for a targeted screen (Fig. S1, A and B).

**Figure 1.**
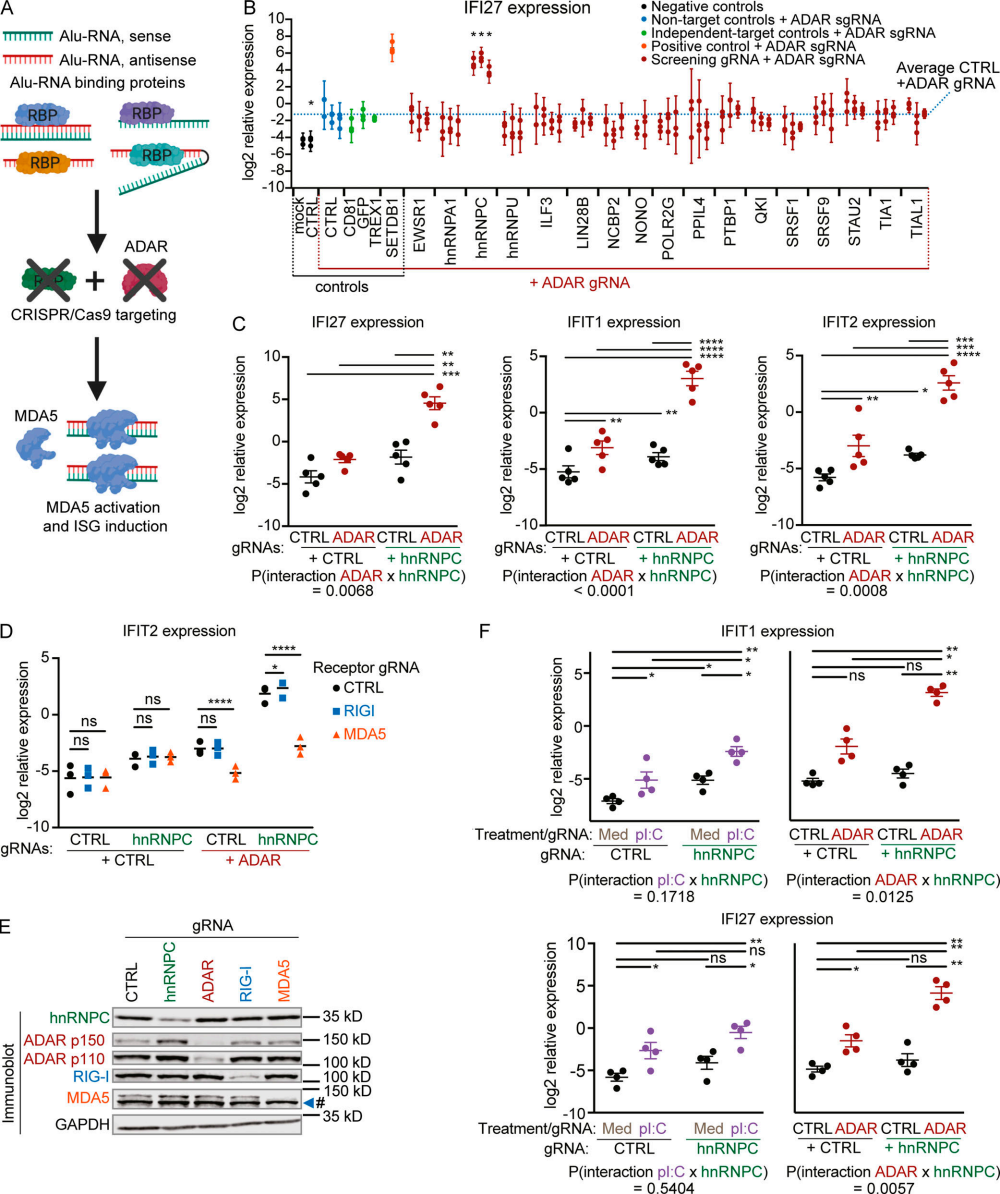
**hnRNPC deficiency synergizes with ADAR deficiency to induce ISGs.(A)** Schematic illustration of screen. **(B)** IFI27 expression in RBP and ADAR CRISPR/Cas9–targeted THP-1 estimated by qPCR. Each set of aligned circles represents unique combinations of RBP targeting + ADAR gRNA (two to four individual gRNAs per RBP). Log2 expression from three independent experiments is reported. Bars indicate 95% confidence interval around mean. * indicates FDR-adjusted P value ≤ 0.05. Successful CRISPR/Cas9 targeting was not confirmed at this stage. **(C, D, and F)** Relative expression of the indicated ISGs estimated by qPCR 5 d after nucleofection of THP-1 with the indicated gRNAs and/or the indicated treatment. Expression was normalized to RPL36 (C) or CASC3 (D and F) expression, log2 transformed, and analyzed by repeated-measures two-way ANOVA; P values for individual comparisons were determined by Sidak’s (C and F) or Bonferroni’s (D) post hoc test. *, P ≤ 0.05; **, P ≤ 0.01; ***, P ≤ 0.001; ****, P ≤ 0.0001. Individual symbols are replicates from five (C), three (D), or four (F) independent experiments; mean (D) or mean ± SEM is depicted (C and F). P values for interaction between ADAR and hnRNPC targeting (C and F) or hnRNPC targeting and pI:C treatment (F) are indicated. Cells were nucleofected with three gRNAs per condition in the indicated combinations, targeting hnRNPC, ADAR, RIG-I, or MDA5. **(E)** Western immunoblot of THP-1 as in D, nucleofected with hnRNPC, ADAR, RIG-I, or MDA5 gRNAs for 4 d. One representative of three experiments. # indicates a nonspecific protein band detected by MDA5 antibody. CTRL, nontarget control; Med, medium; sgRNA, single-guide RNA.

In establishing a screening strategy, we selected the myeloid cell line THP-1 for its robust MDA5-dependent response to transfected polyinosinic:polycytidylic acid (pI:C; data not shown) and its weak IFN response to ADAR inactivation (data not shown), which suggested a putative alternate mechanism for regulation of dsRNA-induced sterile inflammation. To limit technical artifacts resulting from CRISPR/Cas9 DNA damage, which can result in STING activation (Li and Chen, 2018), we generated Cas9-transgenic STING-deficient THP-1 cells (referred to as THP-1 cells unless otherwise indicated). As our preliminary Alu-RBP screens achieved only weak ISG signals (data not shown), we conducted a modifier screen, evaluating the combination of Alu-RBP and ADAR targeting. Endogenous induction of ISGs was measured after 5 d (Fig. 1 A). Histone-lysine N-methyltransferase SETDB1 targeting, which increases dsRNA expression by epigenetically de-repressing endogenous retrovirus- and large interspersed nuclear element–derived RNA, was used as a positive control (Cuellar et al., 2017). Of the 17 Alu-RBPs and four controls targeted, only hnRNPC showed a strong induction of ISGs, measured by the up-regulation of interferon α-inducible protein 27 (IFI27), mitochondrial, IFN-induced protein with tetratricopeptide repeats 1 (IFIT1), and IFIT2 relative to ADAR singly targeting (Fig. 1 B; and Fig. S1, C and D).

**Figure S2.**
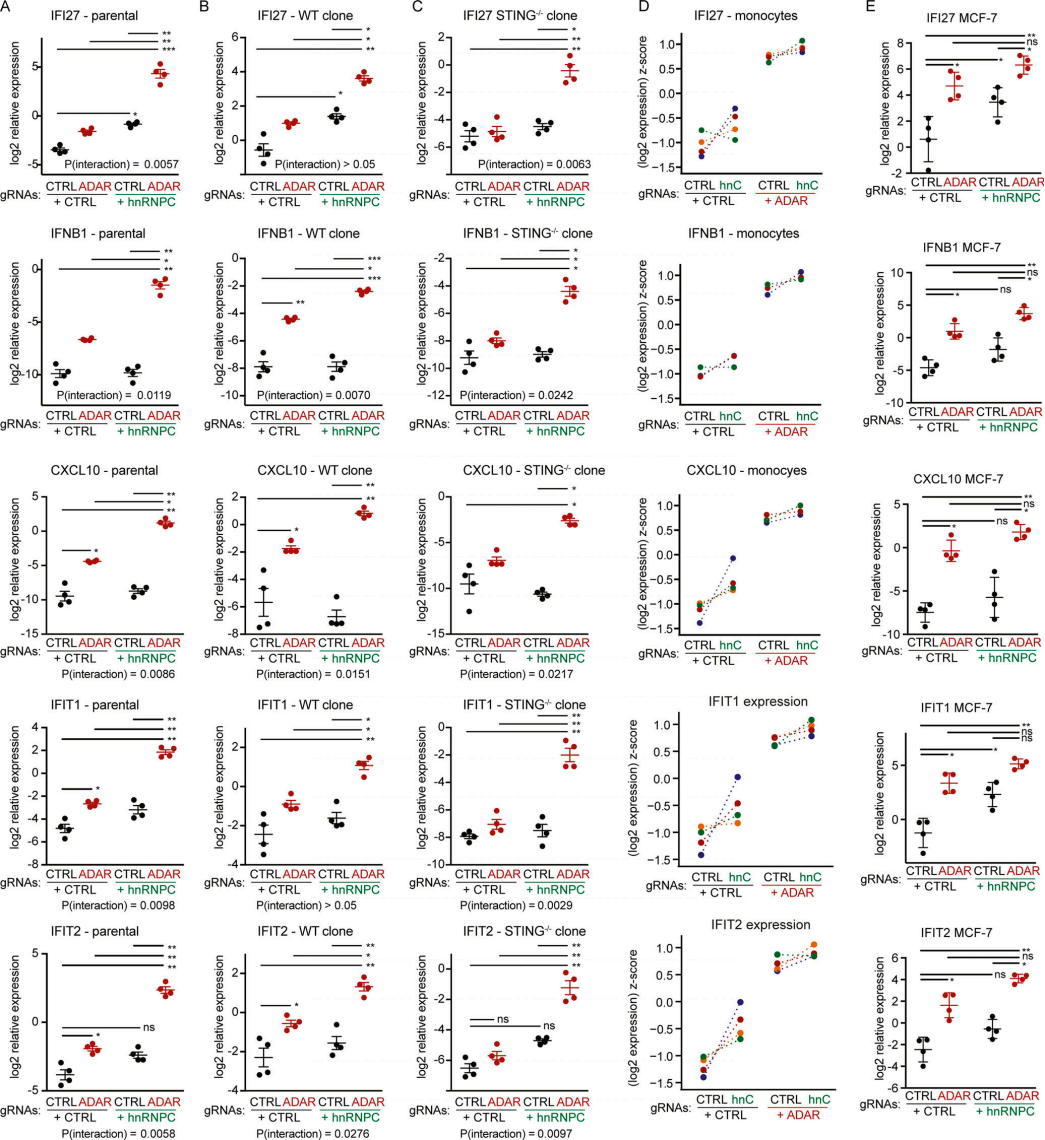
**Validation studies in further cell lines and primary cells.(A–E)** Cells were nucleofected as in Fig. 1 C (A–C) or nucleofected with ribonucleoparticles with recombinant Cas9 and the indicated gRNAs (D and E), and normalized expression of the indicated genes on day 5 (A–C and E) or day 4 (D) is reported. Parental polyclonal WT Cas9-transgenic THP-1 cell line (A), a WT clone (B), a distinct STING-deficient clone (C), or MCF-7 (E) was used. Expression was normalized to RPL36 expression (A–C) or CASC3 expression (D and E), log2-transformed, and then analyzed by repeated-measures two-way ANOVA and P values for individual comparisons determined by Tukey’s post hoc test. *, P ≤ 0.05; **, P ≤ 0.01; ***, P ≤ 0.001 (A–C and E). Individual symbols are replicates from four independent experiments each; mean ± SEM is depicted. **(D)** Relative expression of the indicated genes in monocytes/early macrophages from four independent healthy donors 5 d after nucleofection of the indicated gRNAs. Data are displayed as donor-wise z-scores of log2-transformed relative expression; colors indicate individual donors. CTRL, nontarget control; hnC, hnRNPC.

Independent experiments using one of the hnRNPC-targeting guide RNAs (gRNAs) in the screen and a distinct ADAR gRNA showed successful targeting of hnRNPC and ADAR, as well as up-regulation of ADAR protein upon hnRNPC deficiency (Fig. S1 E). Furthermore, we observed modest ISG regulation by single gene deletion but found a statistically significant synergistic effect of the combined deficiency compared with the combined effects of individual deficiencies (Fig. 1 C; hnRNPC × two-way ANOVA P values for interaction between ADAR targeting and hnRNPC targeting). We confirmed the synergistic ISG induction in (i) the parental WT Cas9-transgenic cell line; (ii) a WT single-cell clone; and (iii) an independent STING-deficient THP-1 line (Fig. S2, A–C). Notably, the synergistic ISG induction was dependent on MDA5 expression and showed no reduction upon RIG-I inactivation (Fig. 1, D and E). We also confirmed a modest induction of ISGs in primary monocytes when hnRNPC was targeted; however, results were variable and were dominated by a strong response to ADAR deficiency. As a result, we could detect an additive (but not synergistic) effect of combined hnRNPC and ADAR targeting in two of four donors (Fig. S2 D). Furthermore, a previous study had described ISG induction in the breast cancer cell line MCF-7 targeted by hnRNPC RNA interference, which could be mildly enhanced by ADAR targeting (Wu et al., 2018). In our hands, targeting either gene using CRISPR/Cas9 led to ISG induction, with stronger ISG induction following ADAR targeting and the combined deficiency resulting in a robust additive effect (Fig. S2 E).

To exclude up-regulation of the dsRNA-sensing machinery as the cause for synergistic ISG induction, we assessed pI:C–mediated MDA5 activation in the presence or absence of hnRNPC. To exclude possible confounding effects of RIG-I, we generated RIG-I–deficient cells and targeted hnRNPC. On day 4, we transfected low-dose pI:C for 24 h. Notably, we did not observe synergistic ISG induction (Fig. 1 F; hnRNPC targeting × pI:C interaction two-way ANOVA P values >0.05). We therefore concluded that synergistic ISG induction was due to changes in the abundance of endogenous dsRNA ligands and not a result of altered baseline activity of the MDA5 pathway. These screening and confirmation studies identified hnRNPC as a novel RNA-binding protein (RBP) responsible for control of endogenous dsRNA, which in some conditions compensated for ADAR deficiency.

**Figure 2.**
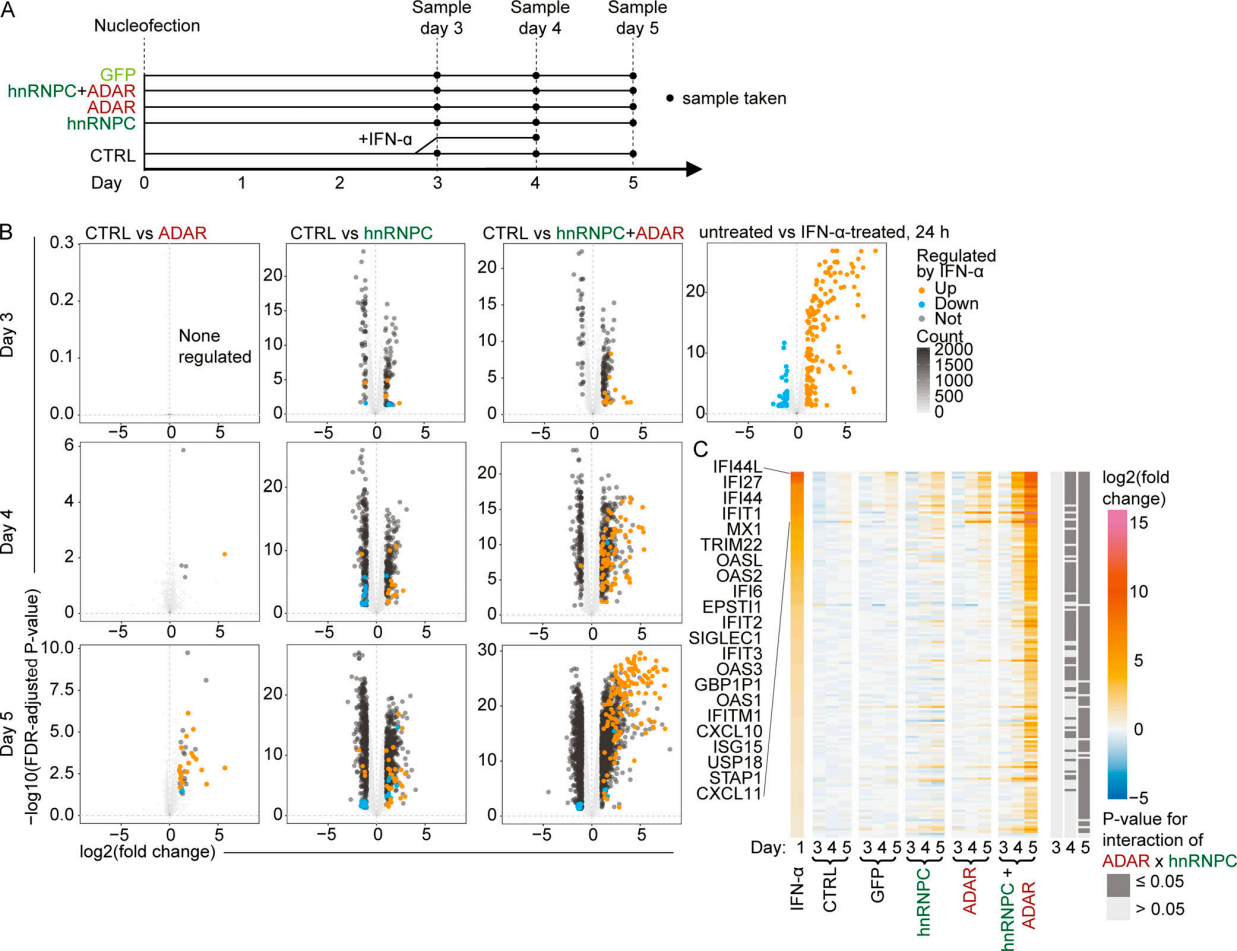
**RNA-seq analysis of gene expression confirms global ISG induction.(A)** Schematic illustration of RNA-seq experimental design: THP-1 was nucleofected with gRNAs on day 0, and cells were harvested on days 3, 4, and 5. In addition, nontarget gRNA nucleofected cells were treated with IFN-α on day 3–4 for 24 h. **(B)** Differential expression analysis of RNA-seq data of THP-1 on day 3–5; comparisons are indicated. IFN-regulated genes are indicated (fold change ≥ 2 and FDR-adjusted P value ≤ 0.05). Only genes differentially regulated in the specific comparisons are displayed; all else are indicated by two-dimensional–density plotting. Correlation coefficient R between IFN-α–regulated genes in IFN-α–treated and ADAR+hnRNPC gRNA nucleofected cells on day 5: 0.889. **(C)** Heatmap of expression of IFN-regulated genes, displayed as fold-change relative to nontarget control (CTRL), ordered by fold-change in IFN-α–treated cells. Top IFN-α–induced genes are indicated. Dark gray annotation bars indicate significant ADAR × hnRNPC interaction (FDR-adjusted P value ≤ 0.05) in factorial analysis on days 3, 4, and 5 each. **(B and C)** Data are from three independent replicates.

To assess the global impact of hnRNPC and ADAR deficiency, we performed RNA sequencing (RNA-seq) using THP-1 cells, evaluated 3, 4, and 5 d after nucleofection (Fig. 2 A). As a positive control, we included recombinant IFN-α for 24 h (Fig. 2 A). We confirmed modest up-regulation of IFN-α–induced genes in singly deficient cells and robust induction in combined hnRNPC+ADAR–targeted cells, with increasing differential expression over the 3-d time course (Fig. 2 B and Data S1). To determine if these IFN-α genes were synergistically induced in the double knockout, we again assessed a statistical interaction between hnRNPC and ADAR targeting for these 158 ISGs by factorial design analysis and found statistically significant interactions for 0, 87, or 147 on day 3, 4, or 5, respectively (false discovery rate [FDR]-adjusted P[interaction] ≤ 0.05; Fig. 2 C and Data S2). Using this RNA-seq dataset, it was possible to explore how hnRNPC could prevent ISG induction.

### hnRNPC suppresses the incorporation of Alu-containing intronic RNA

**Figure S3.**
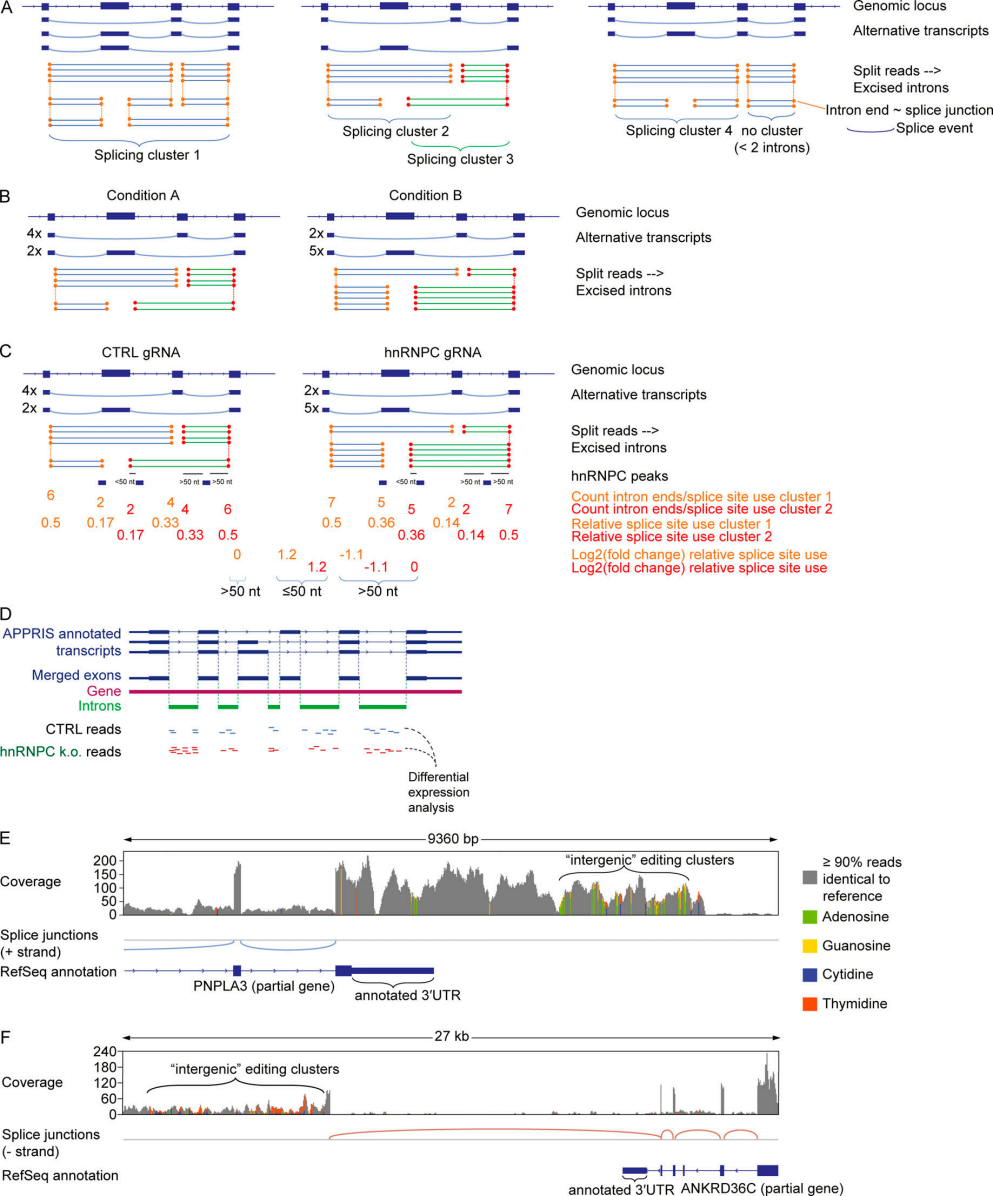
**Alu-containing introns are dysregulated upon hnRNPC deficiency and examples of intergenic annotated editing clusters.(A)** Examples of splicing clusters as defined by LeafCutter. Excised introns are deduced from split RNA-seq reads. Introns sharing ends (~splice sites, circles) are clustered, and clusters containing only one intron are excluded (as shown right). Alternatively spliced transcripts can result in one (left) or multiple (center) splice clusters. Adapted from Li et al. (2017). **(B)** Differential splicing is deduced from differential relative counts of excised introns. Both clusters in this example are differentially spliced. **(C)** Schematic illustration of relative splice site use quantification. Splice site use was estimated by aggregating intron counts sharing the same intron end; relative counts per cluster were calculated, and fold change between nontarget control and hnRNPC gRNA nucleofected THP-1 was determined. **(D)** Schematic illustration of differential expression analysis of intronic regions. Exons of APPRIS-annotated transcripts were merged and subtracted from the respective genes to yield intronic regions. RNA-seq reads mapping to these introns were subjected to intron-wise differential expression analysis. **(E and F)** Exemplary raw coverage of RNA-seq reads and splice junctions estimated from split reads at the PNPLA3 locus (E) and the ANKRD36C locus (F). Analysis shown for cells 4 d after nucleofection with nontarget control (E and F). RefSeq annotation is shown below. Bars are average coverage; gray: <10% reads differ from the reference sequence; colored bars indicate ≥10% of reads differ from reference. Green, adenosine; yellow, guanosine; blue, cytidine; orange, thymidine. Note that the gene in F is transcribed in (−)−strand direction: A-to-I editing appears as C-to-T transition. One representative of three. CTRL, nontarget control; k.o., knockout.

**Figure 3.**
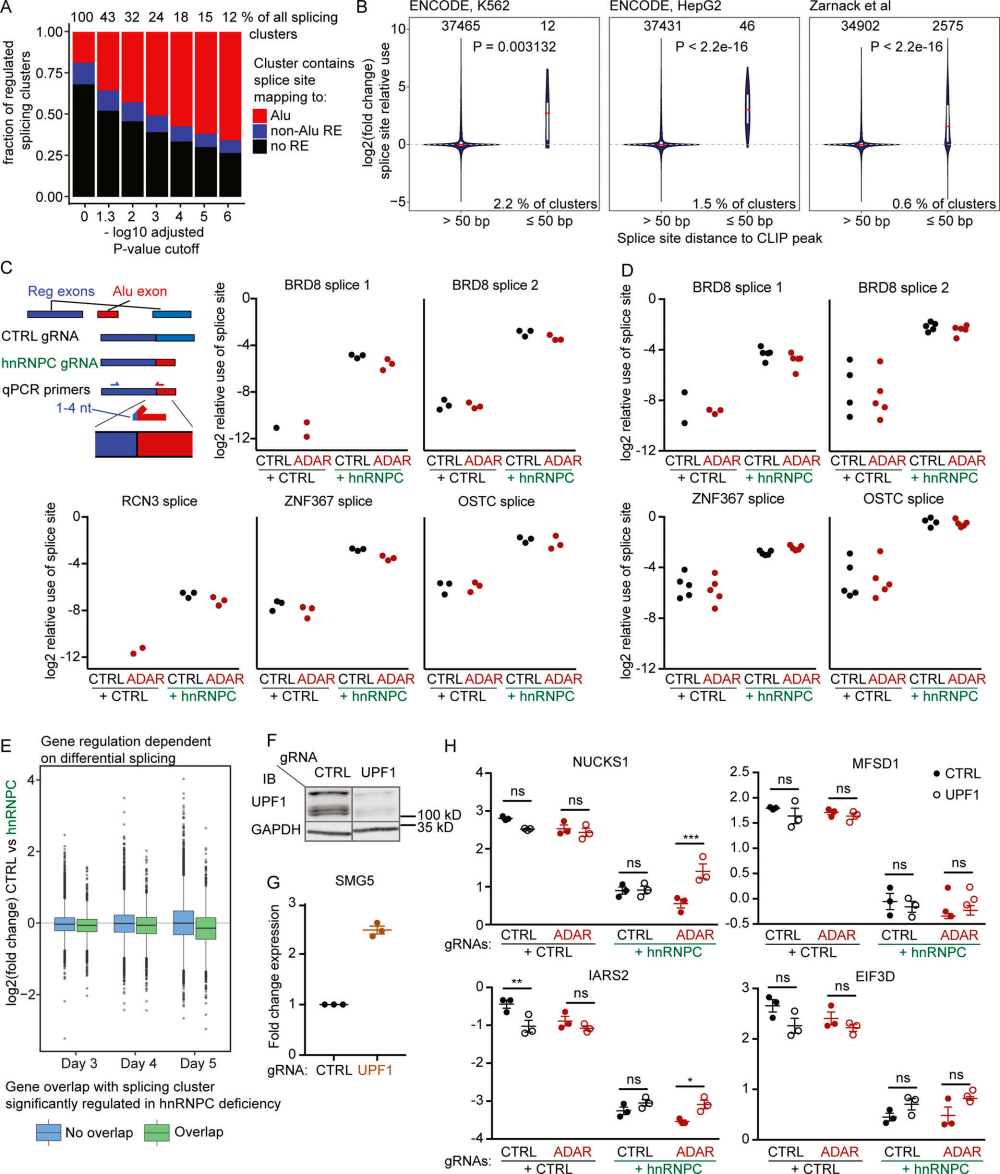
**Differential splicing and effects of NMD in hnRNPC deficiency.(A)** Differential splicing between nontarget control (CTRL) and hnRNPC gRNA nucleofected THP-1 was analyzed as indicated in Materials and methods. Displayed is the relative number of differentially spliced clusters as a function of the FDR-adjusted P value cutoff. Clusters were stratified by the mapping of at least one splice site to one Alu element (red, Alu); no site mapping to Alu, but at least one site mapping to any other RepeatMasker element (blue, non-Alu RE); or no site mapping to any RepeatMasker element (black, no RE). Fractions of differentially regulated splice clusters relative to all clusters for each cutoff are indicated above the bars. **(B)** Relative use of splice sites within significantly regulated splice clusters (P ≤ 0.001), stratified by distance to the next CLIP peak as discovered by the ENCODE project or Zarnack et al. (2013). White bars indicate 25–75 percentile ranges; red line indicates median. P value for Wilcoxon’s rank sum test is indicated. Percentages indicate fractions of CLIP clusters that have ≤50-nt distance to splice junctions contained within significantly regulated splice clusters as defined above. **(A and B)** Data are from three independent experiments. **(C and D)** Confirmation of splice events in THP-1 (C) and MCF-7 (D). To achieve specificity, one primer was designed to match unique exonic sequences, and the second primer was designed to span the splice site with 1–4 nt matching the exon end. Individual symbols are replicates from three (C) or five (D) independent experiments. Missing circles, none detected. Data were normalized to CASC3 expression. **(E)** Log2 fold-change expression of genes upon hnRNPC deficiency on day 3–5, stratified by their genomic overlap with the location of a differentially regulated splice cluster (P ≤ 0.001). Data are from three independent experiments. **(F)** Western blot of CTRL or UPF1 gRNA-targeted cells. One representative of three experiments. WT and UPF1-targeted samples are from the same blot; irrelevant lanes were removed. IB, immunoblot. **(G)** Relative expression of SMG5 in CTRL or UPF1 gRNA-targeted THP-1. Expression was normalized to CASC3. Individual symbols are replicates from three independent experiments; mean ± SEM is depicted. **(H)** Expression of a selection of Alu-exon–containing transcripts that are down-regulated upon hnRNPC deficiency. Expression was normalized to CASC3 expression, log2 transformed, and analyzed by repeated-measures two-way ANOVA, and P values for individual comparisons were determined using Sidak’s post hoc test. Individual symbols are replicates from three independent experiments; mean ± SEM is depicted. *, P ≤ 0.05; **, P ≤ 0.01; ***, P ≤ 0.001. Probes were designed to not span the intron containing the Alu-exon. Closed circles, nontarget control gRNA; open circles, UPF1 gRNA.

hnRNPC had been shown to bind U-rich regions in antisense Alu-RNA, in turn masking cryptic splice sites and preventing incorporation of intronic Alu-RNA into mature transcripts (Zarnack et al., 2013). Furthermore, a recent study showed that hnRNPC deletion in human breast cancer cell lines led to splicing errors and RIG-I–dependent ISG production that was due to exposure of dsRNA-containing intronic products of the nonsense-mediated decay (NMD) machinery (Wu et al., 2018). Using the LeafCutter computational package as an annotation-independent differential splicing discovery tool (Li et al., 2017), we could indeed confirm widespread dysregulated splicing. Moreover, LeafCutter-defined splicing clusters (schematic representation in Fig. S3, A and B), which contain splice sites that map to Alu elements, were enriched among differentially spliced clusters (Fig. 3 A). Furthermore, splice sites from significantly regulated splice clusters and in close proximity (≤50 bp) of hnRNPC CLIP peaks from the ENCODE database (https://www.encodeproject.org/) as well as from the above-mentioned previous study (Zarnack et al., 2013) showed increased relative use upon hnRNPC deficiency (Fig. 3 B; and Fig. S3 C, schematic illustration). We confirmed splicing dysregulation for several transcripts by quantitative PCR (qPCR) in both THP-1 and MCF-7 (Fig. 3, C and D).

Strikingly, ∼27% of expressed genes overlapped with at least one differentially regulated splicing cluster in hnRNPC deficiency (FDR-adjusted P value ≤ 0.01). Genes overlapping with highly regulated splicing clusters (FDR-adjusted P value ≤ 0.001) showed a shift toward decreased expression upon hnRNPC deficiency (Fig. 3 E). These findings are consistent with the engagement of NMD in differentially spliced genes (Attig et al., 2016). It was previously described that NMD in hnRNPC single deficient cells primarily affects genes with small inclusions of Alu sequences. To confirm NMD targeting, we targeted UPF1, an RNA helicase essential for functional NMD (Fig. 3 F; Kim and Maquat, 2019). We measured the expression of SMG5, which is a typical target of NMD during the steady state (Yepiskoposyan et al., 2011), as well as four genes that showed reduced expression in hnRNPC in the RNA-seq and evidence of inclusion of small Alu exons by qPCR (Fig. 3, G and H). While we could confirm decreased expression of all four genes with Alu inclusions, overall, UPF1 targeting had modest effects on the expression of SMG5 and of three of the four putative hnRNPC-knockout–induced NMD targets (Fig. 3, G and H). However, in all cases a trend toward increased expression could be confirmed, indicating contribution of NMD in transcript regulation during hnRNPC expression.

**Figure 4.**
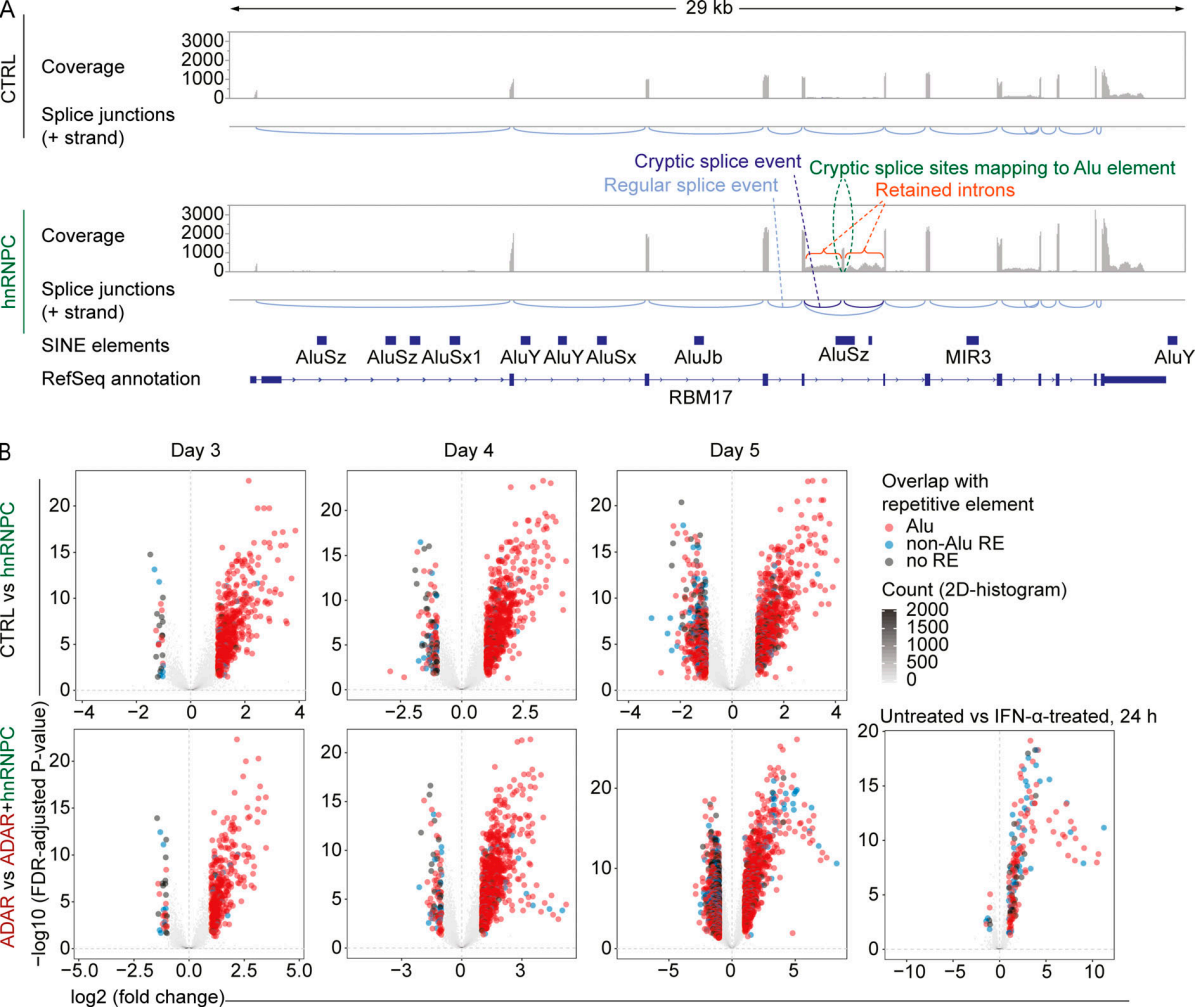
**Dysregulation of introns in hnRNPC-deficient THP-1.(A)** Raw coverage of RNA-seq reads and splice junctions estimated from split reads at the RBM17 locus (+ strand is shown only); analysis shown for cells 4 d after nucleofection with nontarget control (top) or hnRNPC gRNA (bottom). Canonical splice junctions shown in light blue, hnRNPC deficiency–dependent splice junctions in dark blue. One representative of three. **(B)** Differential expression analysis of intronic regions either of cells treated with recombinant IFN-α for 24 h (bottom) or nucleofected with control gRNA (CTRL), hnRNPC gRNA, ADAR gRNA, or both ADAR and hnRNPC gRNA for 3–5 d as indicated in Materials and methods. Comparisons are indicated (as in Fig. 2 B); data are from three independent experiments. 2D, two-dimensional.

While exploring potential sources of dsRNA introduced by hnRNPC deletion, we manually inspected differentially spliced clusters and found expression of long intron fragments that could be incorporated into mature RNAs, as highlighted by a representative example in RBM17 (Fig. 4 A). We hypothesized that the intronic sequences incorporated into mature transcripts could result in the inclusion of inverted repeat-Alu elements as a source of dsRNA. Therefore, we quantified the expression of intronic sequences, as estimated by RNA-seq read counts mapping to intronic regions. To avoid errors in annotation, we derived introns from dominantly expressed transcripts that were annotated within APPRIS (Annotation of Principal and Alternative Splice Isoforms), a well-curated resource (Fig. S2 D; Rodriguez et al., 2013). Differential expression analysis of RNA derived from these APPRIS-annotated intronic regions confirmed increased abundance of Alu-containing introns as described earlier (Wu et al., 2018; Attig et al., 2016; Fig. 4 B and Data S3). We found that this regulation was invariant in ADAR-expressing and ADAR-deficient cells, and excluding a role for an IFN feedback loop, we demonstrated no enrichment for Alu-containing introns in IFN-α–treated cells (Fig. 4 B).

**Figure 5.**
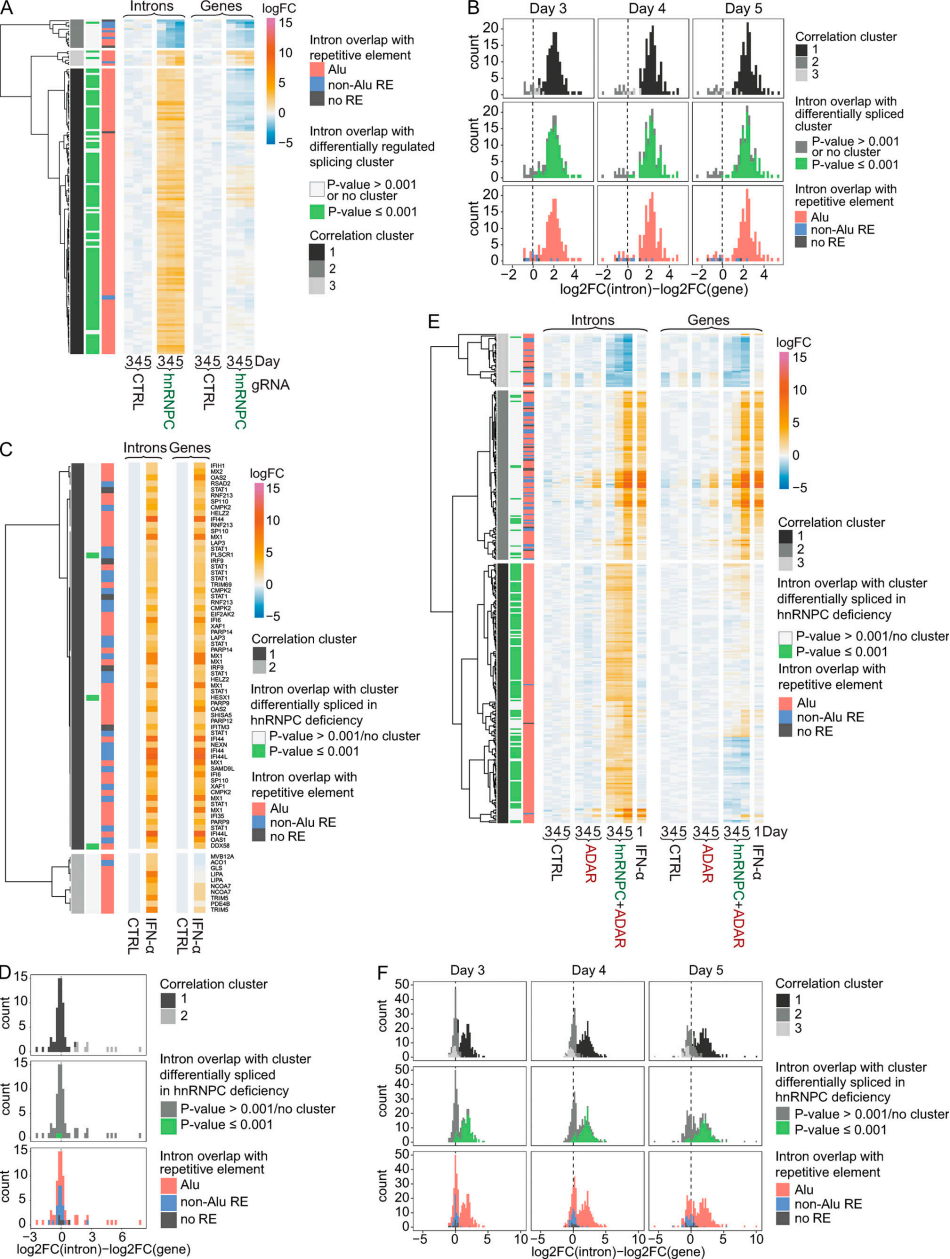
**Splicing-dependent intron dysregulation is independent of gene expression.(A, C, and E)** Heatmap of expression of differentially regulated introns (left, fold change ≥4, FDR-adjusted P value ≤ 0.01 in any per-day pairwise comparison) and their respective genes (right) in nontarget control (CTRL) and hnRNPC gRNA nucleofected cells (A), untreated and IFN-α–treated cells (C), or nontarget control, ADAR gRNA, or ADAR and hnRNPC gRNA doubly nucleofected cells (E). Introns or exonic counts aggregated at the gene level are shown as average log2 (fold change) from three independent experiments, relative to average expression in CTRL nucleofected cells across all days. Data were clustered by correlation. Annotations left of heatmaps indicate correlation cluster (left), overlap of intron with differentially regulated splicing cluster (as in Fig. 3 A; center), or overlap with an Alu element, no Alu but another repetitive element, or no element (right). **(B, D, and F)** Stacked histograms of the difference between the log2-transformed fold change (log2FC) of introns and the corresponding genes as in A, C, and E on days 3–5 as indicated (B and F) or after 24 h of treatment (D). Bar coloring corresponds to annotations in A, C, and E, respectively. logFC, log fold change.

It was important to consider two possible mechanisms that might account for these observations: higher intron expression could be due to increased incorporation into mRNAs; alternatively, it may simply be a reflection of changes in the transcription of the respective gene. Comparing intron regulation with expression changes of respective genes confirmed that hnRNPC deletion results in increased intron RNA abundance in the absence of up-regulated gene expression, indicating increased incorporation of intronic RNA into transcripts (Fig. 5, A and B). By contrast, for most up-regulated introns in IFN-α–treated cells, the respective genes were induced to a similar degree, indicating comparable levels of intron incorporation into differentially regulated transcripts (Fig. 5, C and D). ADAR+hnRNPC double-deficient cells showed a mixed pattern. About half of the differentially expressed introns were regulated in a manner that was independent of gene expression, and these introns contained Alu sequences and overlapped with differentially regulated splicing clusters and therefore were likely targets for cis-regulation by hnRNPC (Fig. 5, E and F). The remaining set of dysregulated introns showed an expression pattern similar to that of their respective genes and were interpreted as being due to the observed differential transcription and likely a result of MDA5 activation. These data definitively established dysregulated splicing in hnRNPC-deficient cells as the determinant of increased expression of Alu-containing introns. See Data S4 for further information about Fig. 5, A, C, and E.

### A-to-I editing cluster-rich RNAs show increased abundance in hnRNPC-deficient cells

**Figure 6.**
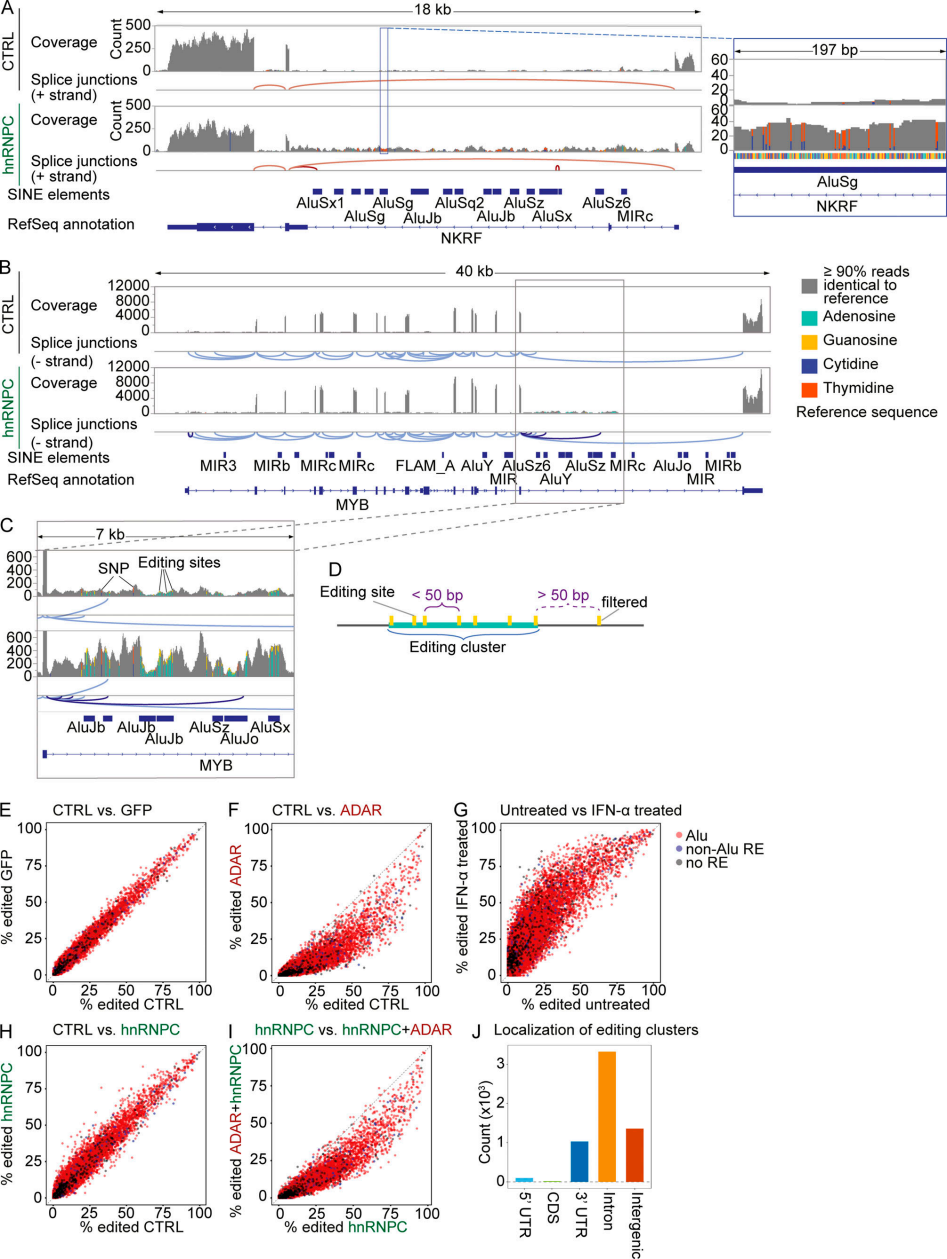
**A-to-I editing sites mainly localize to Alu-elements and introns.(A–C)** Exemplary raw coverage of RNA-seq reads and splice junctions estimated from split reads at the complete MYB locus (A, left, and B) and an intronic region of differential coverage within the gene (A, right, and C); analysis shown for cells 4 d after nucleofection with nontarget control (top) or hnRNPC gRNA (bottom). Data shown are from one representative of three independent experiments. Regular splice junctions shown in light red (A) or light blue (B and C); hnRNPC deficiency–dependent splice junctions in dark red (A) or dark blue (B and C). Bars are average coverage, gray, <10% reads differ from the reference sequence; colored bars indicate ≥10% of reads differ from reference. Green, A; orange, G; red, T; blue, C. Note that nongray bars are highlighted; columns widths are therefore not to scale. One representative of three. Note that NKRF is encoded on the (−) strand, causing A-to-I editing to appear as T-to-C transition. **(D)** Schematic illustration of editing cluster definition and editing clusters. Editing sites with distances 50 bp or lower were clustered; sites with longer distances were excluded from final selection. **(E–I)** Inosine frequencies at individual A-to-I editing sites in cells nucleofected with nontarget control gRNA (CTRL) or targeting GFP, ADAR, and/or hnRNPC, as indicated. Data are from three independent experiments. **(J)** Localization of editing clusters as defined in D.

The most popular model for preventing MDA5 activation suggests a dominant role for the introduction of mismatches in endogenous dsRNA by ADAR-mediated A-to-I editing (Liddicoat et al., 2016). We thus sought to identify stretches of clustered editing in intronic regions up-regulated in hnRNPC-deficient cells. Indeed, while assessing differentially expressed introns, we observed multiple A-to-I editing clusters in a subset of introns, indicating extensive dsRNA formation (Fig. 6, A–C). However, we hypothesized that Alu-containing introns did not comprehensively capture all RNAs containing editing clusters that are differentially expressed in an hnRNPC-deficient state. To discover editing-cluster–rich regions (ECRs) in a transcriptome-wide manner, we employed the SAILOR pipeline, which identifies adenosine-to-guanosine transitions (the result of reverse transcription of inosine bases) in RNA-seq data (Deffit et al., 2017). To remove singleton editing events, we established a filter to capture clusters of multiple edits (five or more), with “cluster” defined by their location within 50 bp of each other (Fig. 6 D). We validated the selected editing sites by comparing editing in WT and ADAR-deficient cells as well as IFN-α–treated (ADAR up-regulated) cells (Fig. 6, E–G). Interestingly, hnRNPC deficiency could lead to both slightly increased and decreased editing in a subset of editing sites. Furthermore, ADAR deficiency combined with hnRNPC reduced editing relative to hnRNPC single deficiency (Fig. 6, H and I). Using all samples as a basis for editing cluster discovery, we found that 57% of the editing clusters were localized to introns, with the remaining mostly shared between 3′ untranslated regions (UTRs; 18%) and unannotated regions (23%; Fig. 6 J). Of the intergenic clusters, the majority were downstream of a 3′ UTR (58.0% ≤5,000-bp distance, 66.8% ≤10,000-bp distance from the end of annotated 3′ UTR) and are likely within read-through or alternatively spliced unannotated 3′ UTRs (Fig. S3, E and F).

**Figure 7.**
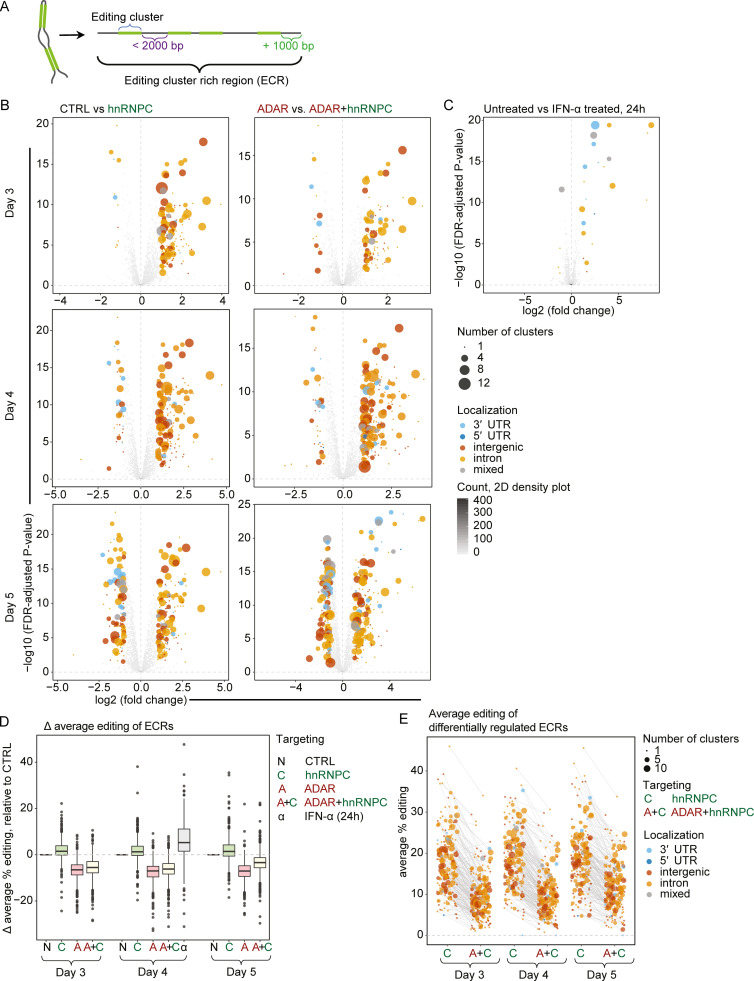
**A-to-I editing cluster–containing RNAs are dysregulated in hnRNPC–deficient THP-1.(A)** Schematic illustration of ECR definition. **(B and C)** Differential expression analysis of ECRs as defined in A on day 3, 4, and 5 after nucleofection as indicated in the Materials and methods; comparisons are indicated. The size of the circles indicates the number of clusters per cluster group; the colors indicate the localization of the clusters contained in the groups. **(D)** Difference of average editing for days 3–5 in ADAR or hnRNPC gRNA nucleofected or IFN-α–treated THP-1, compared with respective nontarget control nucleofected cells. ECRs expressed at ≥4 rpkm across all conditions were considered for analysis. **(E)** Average percent editing of all ECRs on days 3, 4, and 5 in hnRNPC singly and ADAR+hnRNPC doubly targeted cells. Shown are ECRs that were up-regulated on day 4 in hnRNPC-deficient cells. Colors are as in B; size of the circles indicates the number of clusters contained within the ECRs. Lines connect values for individual ECRs between hnRNPC- and hnRNPC+ADAR–targeted cells. **(B–E)** Data are from three independent experiments. CTRL, nontarget control; 2D, two-dimensional.

Direct quantification of editing cluster expression was challenging, since many were localized to Alu elements that often led to multi-mapping RNA-seq reads. To circumvent this problem, we defined ECRs, grouping editing clusters <2,000 bp apart and including 1,000 bp upstream and downstream of the cluster group (strategy depicted in Fig. 7 A). The abundance of multiple intronic and intergenic ECRs was increased in hnRNPC-depleted compared with WT cells, irrespective of ADAR status (Fig. 7 B). These results indicated widespread dysregulation of dsRNAs upon hnRNPC deficiency, while IFN-α treatment as control regulated few ECRs (Fig. 7 C and Data S5). Synergistic ISG induction in ADAR+hnRNPC–deficient cells must depend on both the impaired function of hnRNPC and ADAR. We therefore quantified editing in ECRs and found that, indeed, editing was reduced in ECRs after targeting ADAR, with slightly increased editing upon hnRNPC targeting (Fig. 7 D). Conversely, average editing of ECRs was increased in IFN-α–treated cells, consistent with increased expression of the ISG ADAR. Importantly, ECRs that were differentially regulated upon hnRNPC deficiency showed reduced editing in ADAR-deficient cells, rendering them putative MDA5 ligands (Fig. 7 E and Data S6).

**Figure 8.**
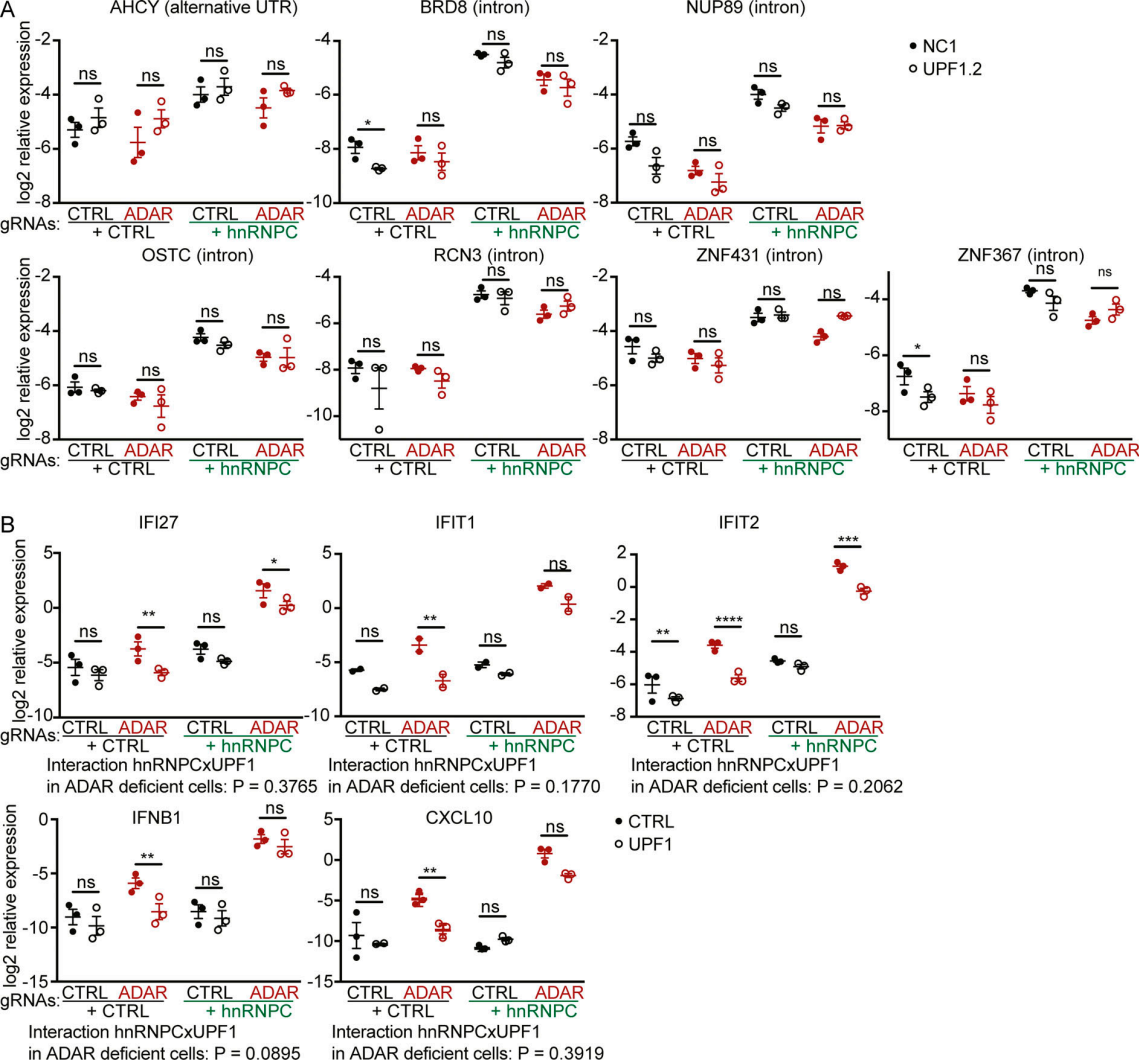
**UPF1 deficiency only mildly affects ECR expression and reduces ISG expression upon ADAR deficiency.(A and B)** Expression of a selection of ECRs (A) or ISGs (B) in THP-1 nucleofected with ADAR, hnRNPC, and/or UPF1 gRNA for 5 d. Expression was normalized to CASC3 expression, log2-transformed, and analyzed by repeated-measures two-way ANOVA and P values for comparisons between CTRL and UPF1 gRNA nucleofected cells determined using Sidak’s post hoc test. *, P ≤ 0.05; **, P ≤ 0.01; ***, P ≤ 0.001; ****, P ≤ 0.0001. Individual symbols are replicates from three independent experiments. CTRL, nontarget control; 2D, two-dimensional.

While many transcripts with small Alu inclusions were targeted by NMD, Attig et al. (2016) indicated that expression of transcripts with long intron inclusions, such as those containing ECRs in hnRNPC-deficient cells, was barely affected. Therefore, we quantified ECR expression in UPF1-targeted cells by qPCR. We confirmed up-regulation in hnRNPC-targeted cells as indicated by RNA-seq but did not observe major effects of UPF1 targeting on ECR expression (Fig. 8 A). Furthermore, we measured ISG expression in cells targeted for hnRNPC, ADAR, and/or UPF1. Unexpectedly, we found that UPF1 targeting led to significant reduction of ISG expression in ADAR singly deficient cells, leading to comparable reduction of ISG expression in ADAR/hnRNPC doubly deficient cells (Fig. 8 B). When including only ADAR-targeted samples into the analysis (Fig. 8 B, red circles), we could not detect any statistical interaction between hnRNPC and UPF-1 targeting, indicating that NMD does not meaningfully affect the expression of ECRs relevant for ISG induction.

These analyses indicate a role for hnRNPC in suppressing expression of multiple potential MDA5 ligands that are present within introns, alternatively spliced or elongated 3′ UTRs, and can be rendered highly immunogenic upon ADAR deficiency.

### A-to-I editing cluster-rich RNAs gain access to the cytosol

**Figure 9.**
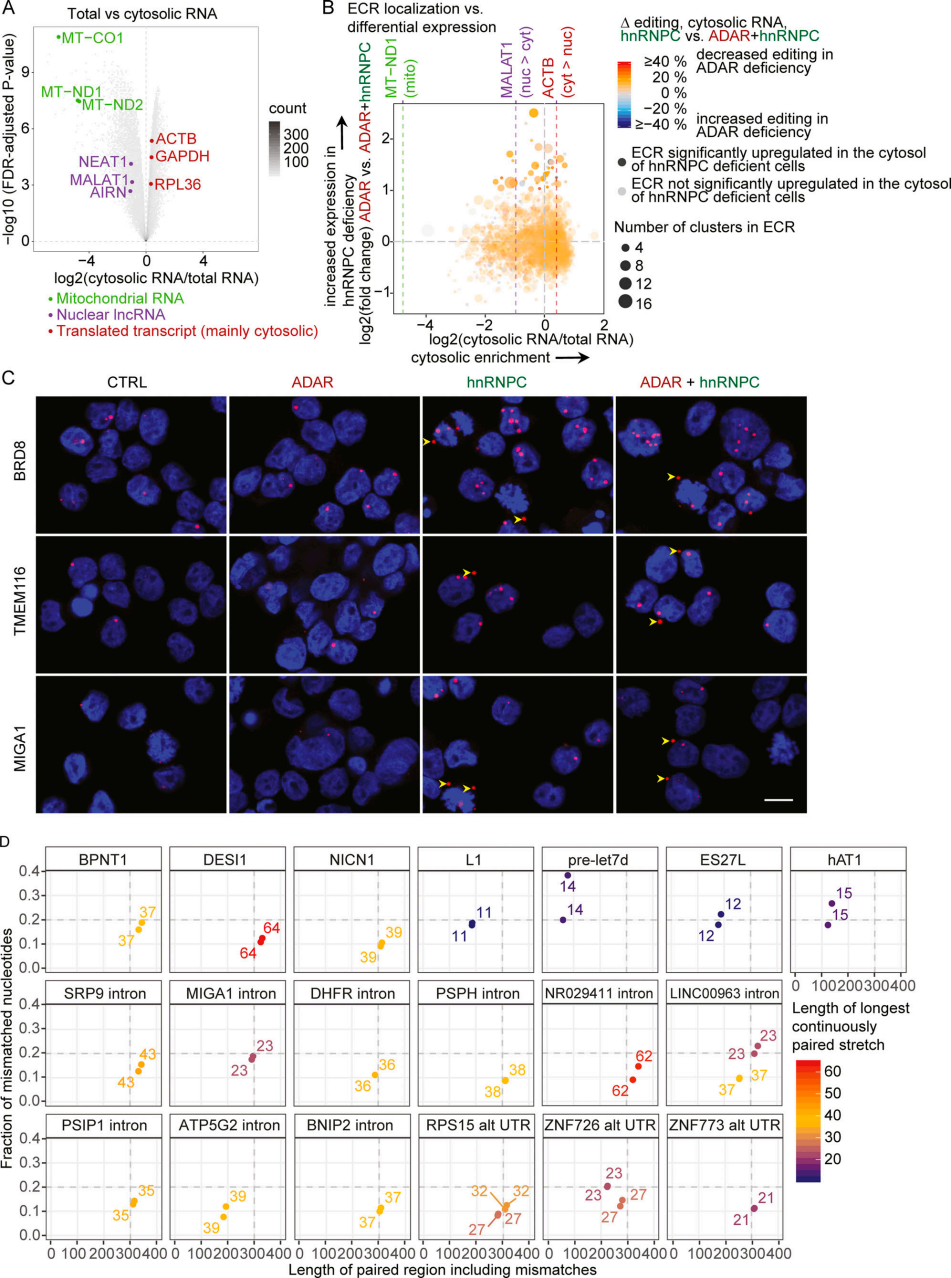
**hnRNPC deficiency–dependent up-regulated editing cluster groups gain access to the cytosol.(A)** Differential expression analysis of genes between total cellular RNA and RNA from cytosolic extracts (see Materials and methods) of THP-1 (WT clone) as indicated in Materials and methods. Mitochondrially encoded genes (green), nuclear lncRNAs (purple), and expressed housekeeping mRNAs are highlighted; all else are displayed as 2D-density plot. **(B)** Four-way plot of log2 (fold-change) expression of A-to-I ECRs. y axis: expression in ADAR gRNA singly versus ADAR+hnRNPC gRNA doubly nucleofected cells on day 4 after nucleofection. x axis: log2 (fold change) between total RNA and RNA from cytosolic extracts. Purple lines indicate values for MT-ND1, MALAT1, and ACTB RNAs as in A. Circle color indicates the average reduction in editing in hnRNPC single versus hnRNPC+ADAR double-deficiency as difference of the average percent editing across all editing sites within each cluster group. Average of two samples per condition. Transparency indicates significant up-regulation in hnRNPC+ADAR over ADAR gRNA nucleofected cells; size of circles indicates number of clusters per cluster group. **(A and B)** Data are from three independent experiments. **(C)** BaseScope analysis (red) of select ECRs in THP-1 with the indicated deficiencies. Arrowheads point to cytosolic transcripts. Nuclei were stained with DAPI (blue). Images are details of images in Fig. S4 B, which are representatives of three images each from two independent experiments. Scale bar, 10 µm. **(D)** Comparison of previously described MDA5 ligands (BPNT1, DESI1, and NICN1) and negative controls (hAT1, L1, pre-let7d, and ES27L) to putative MDA5 ligands within ECRs by indicating length, including mismatches of each strand of the dsRNA stretch, fraction of mismatched nucleotides, and length of the longest uninterrupted dsRNA stretch within putative MDA5 ligands. CTRL, nontarget control; cyt, cytosolic; 2D, two-dimensional; lncRNA, long noncoding RNA; mito, mitochondrially encoded; nuc, nuclear.

**Figure S4.**
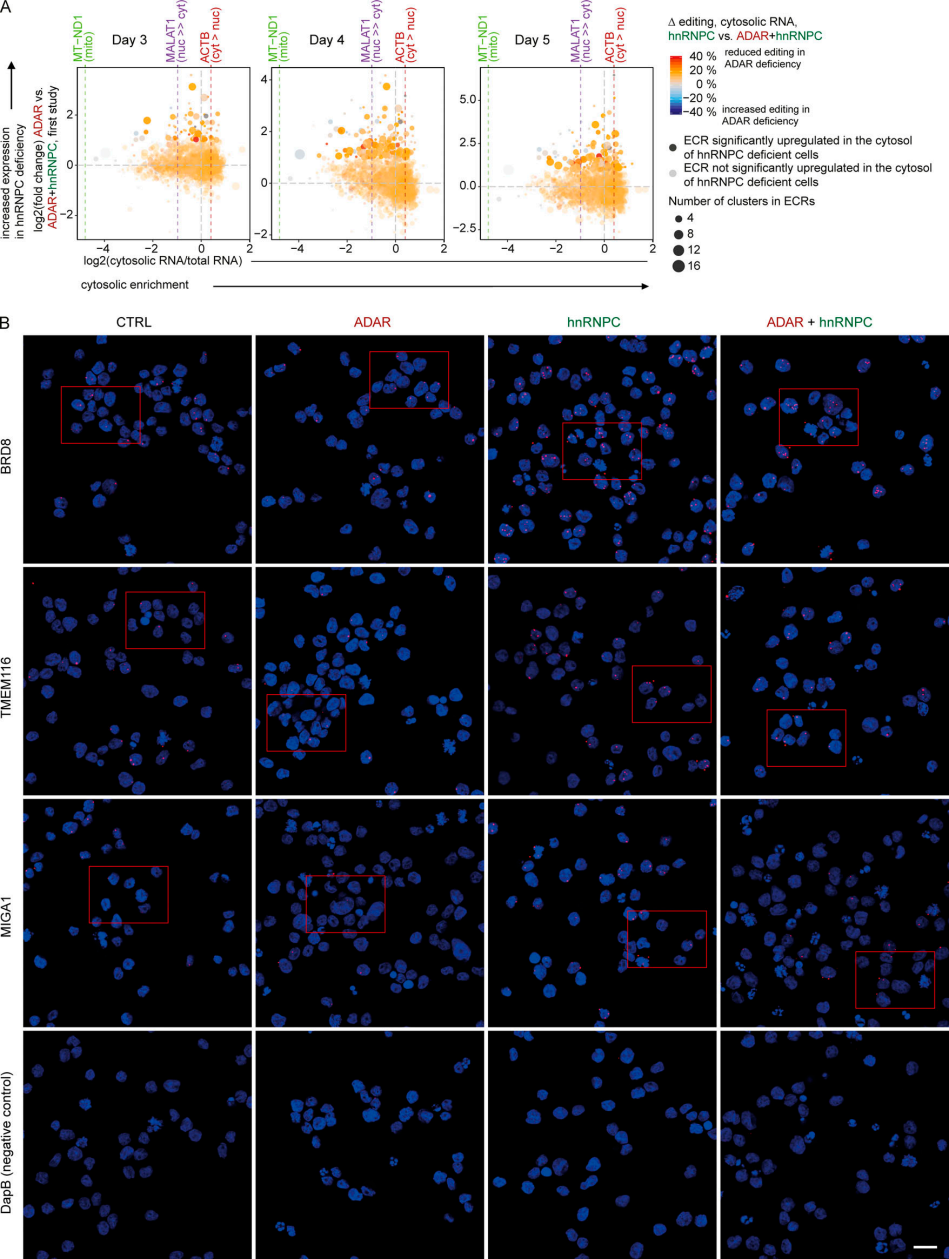
**hnRNPC deficiency–dependent up-regulated ECRs gain access to the cytosol, continued.(A)** Four-way plot of log2 (fold-change) expression of A-to-I editing cluster groups in THP-1. x axis: log2 (fold change) between total RNA and RNA from cytosolic extracts in the second RNA-seq study. Data are from two independent experiments. y axis: expression in ADAR singly versus ADAR+hnRNPC doubly nucleofected cells on day 3, 4, and 5 after nucleofection in the first RNA-seq study. Data are from three independent experiments. Colored lines indicate values for MT-ND1 (mitochondrial), MALAT1 (nuclear), and ACTB (cytosolic) RNAs as in Fig. 9 B. Circle color indicates the average reduction in editing in hnRNPC single versus hnRNPC+ADAR double deficiency as difference of the average percent editing across all editing sites within each cluster group. Average of three (y axis) or two (x axis) samples per condition. Transparency indicates significant up-regulation in hnRNPC+ADAR gRNA over ADAR gRNA nucleofected cells; size of circles indicates number of clusters per cluster group. **(B)** BaseScope staining of select ECRs (red) in THP-1 with the indicated deficiencies. Nuclei were stained with DAPI (blue). Red rectangles represent selected image details shown in Fig. 9 C. Each field of view is representative of six fields of views from two separate experiments. DapB, bacterial gene, negative control. Scale bar, 20 µm. CTRL, nontarget control; cyt, cytosolic; mito, mitochondrially encoded; nuc, nuclear.

In general, retained introns do not gain access to the cytosol (Fazal et al., 2019), and Alu-rich, long noncoding RNAs are more likely to be localized to the nucleus (Lubelsky and Ulitsky, 2018). Nevertheless, a recent study found that Alu-containing introns in hnRNPC-deficient cells were unique in their ability to accumulate in the cytosol (Attig et al., 2016). Therefore, we aimed to test which ECRs might gain access to the cytosol. Cellular fractionation and enrichment of cytosolic RNA was confirmed by the separation of nuclear long noncoding and mitochondrial RNAs from housekeeping mRNAs (Fig. 9 A and Data S7). Using MALAT1 (nuclear), MT-ND1 (mitochondria), and ACTB (cytosolic) RNAs as reference RNAs for the respective subcellular compartments, we found that multiple ECRs, which are dysregulated in hnRNPC-deficient cells, showed greater access to the cytosol compared with MALAT1 (Fig. 9 B, Fig. S4 A, and Data S8). We aimed to confirm cytosolic presence by BaseScope in situ hybridization (ISH). Due to the repetitive nature of ECRs, we were limited in the ECRs we could target. We detected higher expression in hnRNPC deficiency as well as the presence of extranuclear ECRs in a subset of cells, confirming that ECRs are able to be released into the cytosol (Fig. 9 C and Fig. S4 B). To test whether cytosolic putative ligands are comparable to previously described inverted-repeat Alu element (irAlus), which have been described as being able to activate MDA5 filament formation (Ahmad et al., 2018), we identified long paired stretches in a subset of highly up-regulated, likely cytosolic ECRs. We determined their total length, number of mismatches, and length of longest uninterrupted dsRNA, the latter likely constituting the filament formation seed. We found that several of the ECRs harbored paired stretches with very similar properties (length >300 nt, mismatched nucleotide fraction <0.2, and/or longest uninterrupted dsRNA stretch ≥37 bp) as the previously described MDA5 stimulatory irAlus (Fig. 9 D).

**Figure 10.**
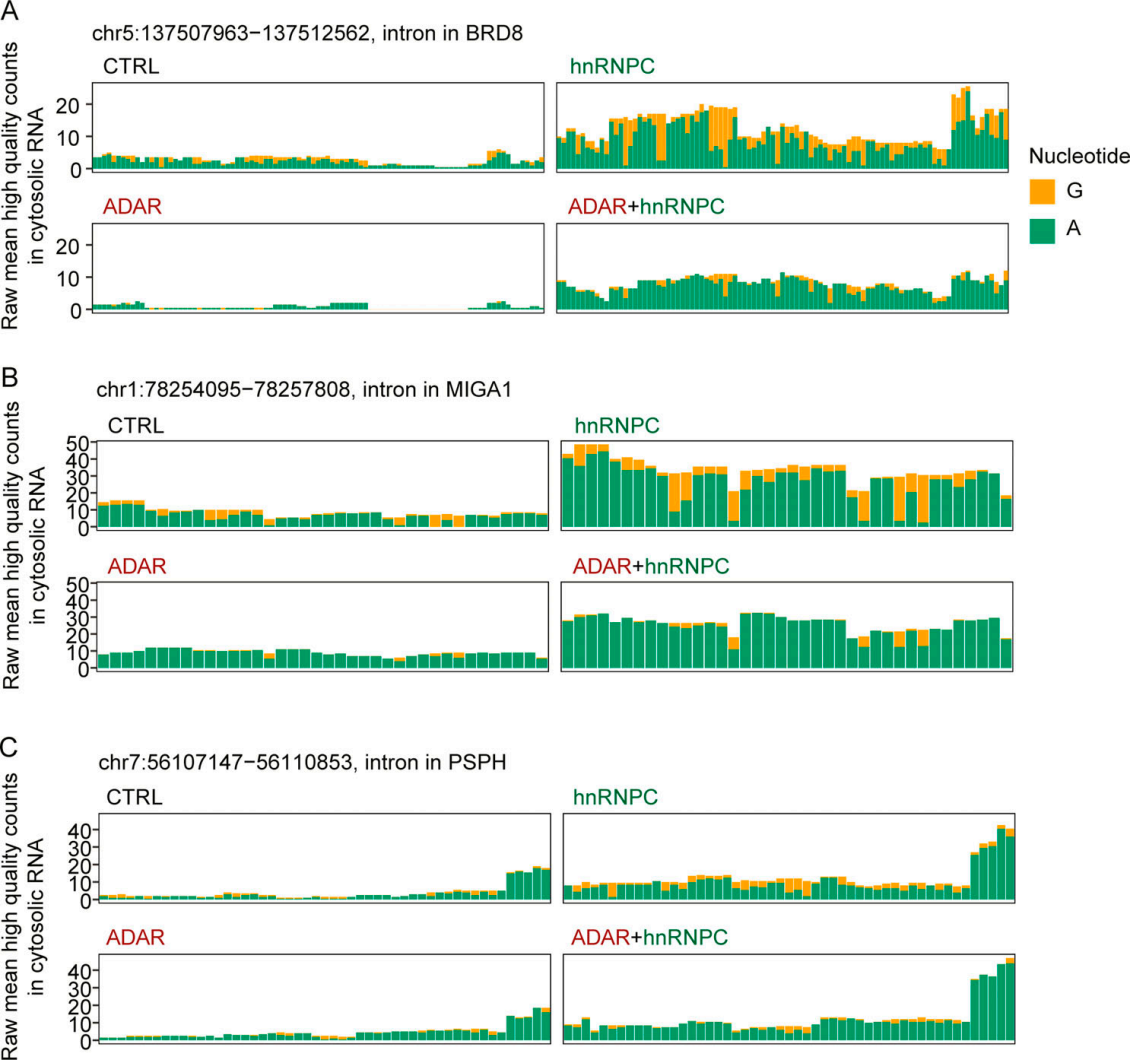
**Editing reduction at individual sites in cytosolic-enriched RNA in ADAR deficiency.(A–C)** Average high-confidence raw counts (*n* = 2) of adenosines (green) and guanosines (= A-to-I edits, orange) at all high-confidence editing sites in exemplary ECRs located to introns in BRD8 (A), MIGA1 (B), or PSPH (C). Shown are data from cytosolic RNA extracts of cas9-transgenic WT THP-1. Deficiencies are indicated. Data are from two independent experiments. A, adenosine; CTRL, nontarget control; G, guanosine.

As only unedited endogenous dsRNAs are believed to activate MDA5, we estimated differential editing of putative ligands in ADAR deficiency. Importantly, the set of putative MDA5 ligands contained multiple editing clusters and showed reduced editing in ADAR-deficient cells, mean of absolute reduction, 12.2%; mean of relative reduction, 53.4% (Fig. 9 B). Furthermore, individual sites within ECRs showed much stronger reduction in editing, especially those with high editing frequencies in ADAR WT cells (Fig. 10). We conclude that hnRNPC protects the host cell from MDA5 ligands that might otherwise have access to the cytosol, establishing a second line of endogenous defense for inverted repeat Alu elements.

## Discussion

**Figure S5.**
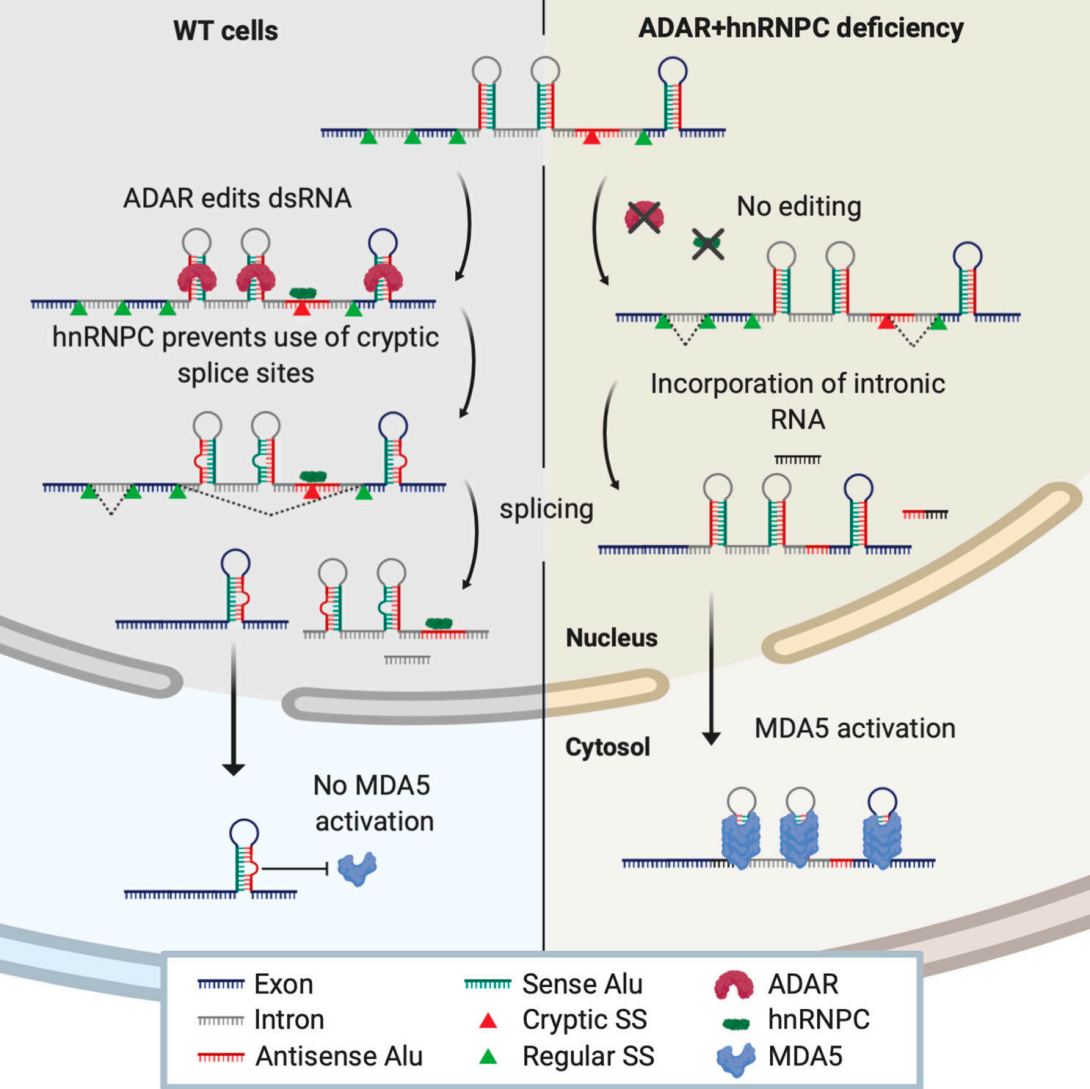
**Schematic illustration of the proposed model.** In WT cells (left), dsRNA (mostly inverted-repeat Alu element RNA) is edited by ADAR. In addition, hnRNPC prevents the use of cryptic splice sites and prevents the incorporation of dsRNA-containing intronic RNA into mature mRNA. Thereby, only edited RNA prevented from activating MDA5 is released into the cytosol. In hnRNPC and ADAR deficient cells (right), dsRNA remains unedited and the use of cryptic splice sites leads to incorporation of intronic RNA into mature transcripts. These contain multple dsRNA stretches capable of activating MDA5. SS, splice site.

In this study, we demonstrated that ADAR and hnRNPC cooperatively prevent autoinflammatory activation of MDA5 by endogenous dsRNA stretches. While ADAR marks endogenous dsRNA structures by editing As-to-Is, thereby reducing their “double strandedness” (Liddicoat et al., 2016), hnRNPC limits MDA5 access to dsRNA derived from introns or intergenic regions’ neighboring genes by preventing cryptic splice site usage (illustrated in Fig. S5). Upon hnRNPC deficiency, ADAR partially compensates for the higher abundance by A-to-I editing, but upon deficiency of both RBPs, an excess of unedited dsRNA-containing transcripts in the cytosol leads to synergistic ISG induction. We demonstrated that these dsRNA-containing transcripts originated from retained introns or intergenic RNAs. Their intergenic localization suggested that they are independently expressed and may also be derived from elongated 3′ UTRs or alternative 3′ UTRs. With respect to the latter class of transcripts, hnRNPC has been described as suppressing the use of distal polyadenylation sites (Gruber et al., 2016) as well as the exonization of intergenic Alu elements, which may result in alternative 3′ UTR usage (Tajnik et al., 2015).

A recent study reported that hnRNPC deficiency induced type I IFN responses in breast cancer cells. These findings indicated a role for RIG-I activation by transcripts that were subject to NMD (Wu et al., 2018). While transcripts with exonized Alu elements in hnRNPC-deficient cells have been shown to be subjected to NMD (Attig et al., 2016), the study did not demonstrate a role for MDA5 (Wu et al., 2018). In our studies, the synergistic ISG response was independent of RIG-I, indicating the possibility that two distinct cellular sensors are able to recognize hnRNPC-regulated endogenous ligands. Furthermore, in contrast with the previous study, UPF1 targeting did not affect ISG expression in hnRNPC singly deficient cells. To our surprise, ISG responses following ADAR deficiency were strongly reduced by UPF1 depletion, which also impacted the ISG response to ADAR/hnRNPC combined deficiency. To our knowledge, the UPF1 dependency of the response to ADAR deficiency has not been previously described and should be followed up in further studies. It remains unclear whether NMD is required for MDA5 ligand activity or whether UPF1 plays a more direct role in MDA5 signaling by, for example, protein–protein interactions. Due to this finding, we could not fully disambiguate the role of NMD in hnRNPC/ADAR deficiency. Nonetheless, the lack of increased ECR expression suggested likely resistance to NMD for the identified putative MDA5 ligands. This conclusion is in accordance with a previous study that described that long introns incorporated during hnRNPC deficiency are not subject to NMD (Attig et al., 2016).

Differences in cytosolic RNA receptor expression could explain differences observed in our study and the study by Wu et al. (2018). We could not exclude, however, activation of further dsRNA receptors or a possible hierarchy to the response, as RIG-I is expressed in THP-1, indicating that the RIG-I–activating capacity of NMD-processed hnRNPC-dependent ligands may be subordinate to the more dominant response induced by MDA5 being engaged by long, unprocessed, unedited Alu-containing dsRNAs. An additional caveat that challenges direct comparison is the technical aspects of genetic perturbation: RNAi in Wu et al. (2018) versus CRISPR/Cas9 targeting in our study, with the latter resulting in more limited off-target effects (Smith et al., 2017).

While we aimed to identify the exact nature of the hnRNPC-limited MDA5 ligands, we faced multiple technical challenges (promiscuous binding of MDA5 to any RNA, mapping challenges for repetitive elements, byproducts in in vitro–generated ligands, and failed cross-linking of MDA5 and its target RNA in eCLIP-like experiments). In fact, the optimal endogenous RNA ligand(s) are still ill-defined. A recent study showing that an editing-insensitive MDA5 mutant can protect irAlu element RNA in vitro was a first step into discovering and characterizing endogenous MDA5 ligands (Ahmad et al., 2018; Mehdipour et al., 2020). However, the true nature of the MDA5 recognition motif remains controversial. While in vitro experiments and crystal structure studies suggest perfect dsRNA longer than 300 bp is a prerequisite for MDA5 activation (Peisley et al., 2011, 2012; Wu et al., 2013), endogenous RNA ligands with these features have not been formally identified. Using acridine orange staining, one study suggested that the main stimulatory components during viral infection are high molecular weight, complex, or even branched RNA structures that are most effective at activating MDA5 during viral infections (Pichlmair et al., 2009). A more recent study suggests that even 300-bp inverted repeat Alu element RNA hairpins, including several mismatches, could activate MDA5 in vitro, which was inhibited by in vitro ADAR editing (Ahmad et al., 2018). These ligands could be identified by an in vitro MDA5 filament formation and RNase protection assay. This study was recently supported by another study that similarly confirmed irAlu RNAs as ligands for WT MDA5 by in vitro RNase protection (Mehdipour et al., 2020). The number of protected irAlu RNAs could be increased upon treatment with the DNA-methyltransferase inhibitor 5-aza-2′-deoxycytidine and/or ADAR deficiency. The increase in irAlu protection by MDA5 upon 5-aza-2′-deoxycytidine treatment was due to an increase in Alu expression driven by demethylated orphan CpG islands, independent of neighboring gene expression. De-repressed irAlu expression led to ISG induction and sensitization of cancer cells to ADAR targeting (Mehdipour et al., 2020). Our data strongly support Alu elements as the main source of potential endogenous MDA5 ligands, since they form the majority of dysregulated MDA5 ligands in hnRNPC deficiency. Independent of hnRNPC targeting, a recent study described spliceosome inhibition in triple-negative breast cancer with MYC-driven hyperactivated transcriptional activity. Similar to the results found in hnRNPC-deficient cells, spliceosome targeting led to release of intronic dsRNA such as irAlus into the cytosol and ISG induction (Bowling et al., 2021).

Our work sheds important light on the regulation of sterile inflammation during malignant transformation. Regarding the regulation of RNA processing, including splicing and 3′ UTR end processing, it is often dysregulated in tumor cells (Obeng et al., 2019). In several studies, this phenomenon has been linked to hnRNPC expression levels. Interestingly, it has been shown that hnRNPC is overexpressed in lung, colon, hepatocellular, and oral squamous cell carcinoma and glioblastoma (Sun et al., 2007; Park et al., 2012; Sebestyén et al., 2016; Huang et al., 2020; Guo et al., 2021), with lower expression associated with better survival (Tremblay et al., 2016; Huang et al., 2020; Guo et al., 2021). Some mechanisms by which hnRNPC might be implicated in malignant transformation have been described, including differential c-myc translation, BRCA1 splicing, and regulation of PDCD4 expression (Kim et al., 2003; Park et al., 2012; Anantha et al., 2013). Our data indicate that increased hnRNPC expression may also be a way of escaping the activation of innate immune receptors under conditions of transcriptional stress during increased proliferation. In many cancers, splicing is dysregulated; therefore, hnRNPC may be overexpressed to compensate and prevent the access of dsRNA to the cytosol (Sveen et al., 2016). In light of the active development of ADAR inhibitors, low hnRNPC expression could serve as a predictive biomarker. Importantly, the sensitization for ADAR deficiency by hnRNPC deficiency was particularly pronounced in transformed cells but not in primary cells, suggesting a putative therapeutic window that could be exploited for systemic use of ADAR inhibitors. As an alternative strategy, our work also elevates tumor-specific targeting of hnRNPC as a means to enhance cancer immunotherapy.

## Materials and methods

### CRISPR targeting of cell lines

To generate protein-deficient cells, Cas9-transgenic THP-1 or MCF-7 cells were nucleofected with hybridized CRISPR RNA (crRNA)+trans-activating crRNAs (referred to as gRNA) as described before (Cuellar et al., 2017). Briefly, 2 d before nucleofection, THP-1 was centrifuged (350 *g*, 5 min, room temperature [RT]), and the pellet was resuspended in RPMI at a density of 0.5e6 cells/ml or confluent MCF-7 split 1:2. For nucleofection, gRNAs were freshly annealed by mixing 100 µM crRNA and 100 µM transactivating crRNA in nuclease-free duplex buffer (IDT), heating to 95°C for 3 min and then cooling to 4°C over the course of 10 min in a PCR cycler. All RPMI recovery medium was incubated at 37°C and 5% CO_2_ for at least 30 min. For MCF-7, gRNAs (3 µl hnRNPC gRNA or control and 1.5 µl ADAR gRNA or control) were mixed and incubated with 1.5 µl recombinant V3 Cas9 protein (IDT) at RT for 10 min, then placed on ice until use. 2e6 THP-1 or MCF-7 per reaction was precipitated for 5 min at 350 rcf and RT, resuspended in PBS, and precipitated again. Then, cells were resuspended in 20 µl P4 primary cell buffer including supplement (Lonza; THP-1) or SG Cell Line buffer including supplement (Lonza; MCF-7). gRNAs (THP-1) or ribonucleoparticles (MCF-7) targeting different genes were nucleofected simultaneously; 2 µl per well of each gRNA (THP-1) or 6 µl premixed ribonucleoparticles (MCF-7) was pipetted into 16–well-strip cuvettes (Lonza), and 20 µl cells was added and carefully mixed. After 5-min incubation at RT, cells were nucleofected using the Lonza 4D-Nucleofector X unit with protocol CM138 (THP-1) or CM137 (MCF-7). For recovery immediately after nucleofection, 100 µl medium was added to the cuvette wells, and the suspension was added to 70 µl medium in a U-bottom 96-well plate. From here, cells were transferred to 1 ml medium per nucleofection and incubated at 37°C and 5% CO_2_ for 3–5 d (THP-1) or 4 d (MCF-7) until harvest for analysis. For generation of STING-deficient single-cell clones, cells were sorted with a FACSAria Fusion cell sorter 1 d after nucleofection of gRNAs. The WT THP-1 clone used in Fig. 9, A and B; Fig. 10; Fig. S2 B; and Fig. S4 A, as well as the WT clone used for BaseScope staining (Fig. 9 C and Fig. S4 B) were generated in parallel by limiting dilution. crRNA sequences are listed in Table S2.

If double-deficiencies were induced in an experiment, single deficiencies were achieved by substituting one of the gRNAs with nontarget control (CTRL). These CTRL additions are implied in the text when mentioning only one gRNA or a single deficiency. Accordingly, targeting gRNAs were substituted for nontargeting controls in triple-targeting experiments. Exact conditions are listed in Table S3. STING-deficient clones were checked for deficiency phenotypically (DNA transfection and lack of IFN-β secretion) and by Western blot.

### pI:C transfection

On day 4 after nucleofection, THP-1 cells were counted, spun down, and concentrated to 1e6 cells/ml, and 5e5 cells per well were seeded in 24 wells without media change. For pI:C transfection, per well 0.8 µl TransIT-LT1 (Mirus Bio) was added to 99.2 µl Opti-MEM and incubated for 5 min at RT. 400 ng (0.4 µl) pI:C was added to 99.6 µl Opti-MEM, mixed with the TransIT LT1/Opti-MEM mix, incubated 20 min, mixed again, and added to the cells. Cells were harvested for qPCR analysis after 24 h.

### CRISPR targeting of primary monocytes/macrophages

Buffy coats were purchased from Vitalant. Leucosep tubes (Greiner Bio-One; 50 ml) were filled with 15 ml Ficoll-Paque PREMIUM and centrifuged for 1 min at 1,000 *g*. Buffy coats were diluted 1:1 with PBS and layered on top of the Leucosep membrane. The gradients were centrifuged for 15 min at 800 rcf and RT, without breaks. The white blood cell layer was aspirated and washed with PBS (centrifugation at 400 rcf, 4°C, 7 min). Red blood cells were lysed for 5 min at RT in ACK red blood cell lysis buffer (163 mM ammonium chloride, 1 mM potassium bicarbonate, and 0.1 mM EDTA, pH 7.4) and washed two or three times with PBS (centrifugation at 400 rcf, 4°C, 5 min). Cells were resuspended in MACS buffer (PBS with 0.5% BSA and 2 mM EDTA) and counted. Monocytes were then isolated according to protocol using the Pan Monocyte Isolation Kit, Human (Miltenyi Biotec). Isolated monocytes were frozen in FCS/10% DMSO for later processing. Cells were resuspended in macrophage-CSF medium (DMEM, high glucose, 10% FBS, 1 × Glutamax [Gibco], 1% Penicillin/Streptomycin, and 10 ng/ml human recombinant M-CSF [VWR]) and incubated for recovery overnight. Then, the cells were detached using Trypsin/EDTA and nucleofected as described in CRISPR targeting of THP-1 cells with the following changes: Per reaction, 3 µl annealed gRNA was complexed with 2 µl TrueCut Cas9 Protein V2 (Invitrogen) by incubating 10 min at RT before nucleofection, 10^6^ cells were nucleofected per reaction, protocol CM137 and P3 primary cell buffer including supplement (Lonza) was used, and cells were nucleofected directly after being added to the gRNA/Cas9 complexes. Cells were resuspended in M-CSF medium at 10^6^ cells/ml and used for analysis 5 d after nucleofection.

### RNA preparation and qPCR analysis

For RNA preparation from THP-1, cells were precipitated (350 *g*, 5 min, RT) and prepared using the RNeasy Mini Kit (Qiagen) according to the manufacturer’s instructions, including the recommended on-column DNase digest. For RNA preparation from primary monocytes/macrophages, cells were detached using Trypsin/EDTA and prepared using the Qiagen AllPrep DNA/RNA Mini Kit. RNA was either directly employed for cDNA synthesis or, to ensure proper detection of secondary-structure-rich RNA, mixed with water for cDNA synthesis, heated to 95°C for 30 s to denature secondary structures, and cooled to 4°C; then, cDNA was synthesized using the iScript cDNA synthesis kit according to the manufacturer’s instructions. cDNA was then diluted 1:20, and Taqman qPCR was performed with the following reactions per well: 5 µl TaqMan Universal PCR Master Mix, 2.5 µl water, 0.5 µl Thermo Fisher Scientific TaqMan Gene Expression Assay (FAM-MGB), and 2 µl diluted cDNA. SYBR green qPCR was performed using 5 µl SYBR Select Mastermix, 0.3 µl primer-mix (10 µM each), 2.7 µl water, and 2 µl diluted cDNA per well. qPCR was performed using a QuantStudio5 using the standard protocol (40 cycles) and relative expression using RPL36 (Fig. 1 C; and Fig. S2, A–C) or CASC3 (all other figures) as housekeeping gene was determined using the comparative cycle threshold (ΔΔCt) method. Thermo Fisher Scientific qPCR assays used in this study were CASC3: Hs00201226_m1, CXCL10: Hs00171042_m1, IFI27: Hs01086373_g1, IFIT1: Hs03027069_s1, IFIT2: Hs00533665_m1, IFNB1: Hs01077958_s1, RPL36: Hs03006033_g1, NUCKS1: Hs01068055_g1, MFSD1: Hs00224178_m1, IARS2: Hs01058378_m1, and EIF3D: Hs01044815_m1. SYBR green primers are listed in Table S4.

### Statistical analysis

RNA levels were measured in technical duplicates. The average of the duplicates was log2-transformed and used as one biological replicate. Biological replicates were derived from experiments conducted on separate days or from separate blood donors. If not stated otherwise, three or more of these log-transformed biological replicates were used as a basis for statistical analysis using Prism 7. For the screen, repeated-measures one-way ANOVA using the results within each biological replicate as repeated measures was conducted, including Geisser-Greenhouse correction. For pairwise hypothesis testing, Benjamini and Hochberg FDR correction was used. For all other qPCR experiments, repeated-measures two-way ANOVA was conducted using the results within each biological replicate as repeated measures. For Fig. 1, C and F; Fig. 3 H; Fig. 8; and Fig. S2, A–C and E, pairwise comparisons were corrected using Sidak’s method, and all possible comparisons were included if not indicated otherwise. For Fig. 1 D, Bonferroni correction was used, and for each hnRNPC/ADAR deficiency combination, RIG-I and MDA5 deficiency were compared only to nontarget control.

### Western blot

Cells were washed with PBS and lysed with RIPA (Sigma) including 1× HALT protease inhibitor (Life Technologies) for 30 min on ice. Lysates were then cleared by centrifugation at 20,000 rcf for 10 min at 4°C, and protein concentrations were determined using the Pierce BCA Protein Assay Kit (Thermo Fisher Scientific). 10–20 µg of protein was separated on a 12–4% Bis-Tris or 20–4% Tris-Glycine polyacrylamide gel. Proteins were transferred to nitrocellulose membranes by standard wet transfer and then briefly washed in Tris-buffered saline (TBS) and blocked in 3% nonfat dry milk (Bio-Rad) in TBS for 1–2 h. The membranes were then washed and incubated with primary antibodies (listed below) in 1% nonfat dry milk in TBS + 0.1% Tween 20 (TBS-T) under shaking at 4°C overnight. Membranes were washed with TBS-T and incubated with secondary antibodies for 1–2 h. Membranes were washed 2× with TBS-T and 2× with TBS, and then fluorescence was detected using a Li-COR Odyssey Fc instrument. Primary antibodies were anti-ADAR: Cell Signaling Technologies 14175; anti-hnRNPC: abcam ab10294; anti-GAPDH: Cell Signaling Technologies 5174; anti–RIG-I: Adipogen AG-20B-0009-C100; anti-MDA5: Enzo Life Sciences ALX-210-935-C100; and anti-UPF1: abcam ab109363; secondary antibodies were Li-COR goat–anti-rabbit IgG or goat–anti-mouse IgG, IRDye 680 RD or IRDye 800 CW conjugates.

### Cell fractionation

Cells were fractionated as described in Baghirova et al. (2015) with the following modifications: cells were collected from suspension, precipitated by centrifugation (350 rcf, 4°C, 5 min), washed with ice-cold PBS, precipitated (350 rcf, 4°C, 5 min), and lysed with lysis buffer A supplemented with 1× HALT protease inhibitor as well as 0.5 U/µl RNase inhibitor (Applied Biosystems) and an increased digitonin concentration (300 µg/ml) for 10 min on an end-over-end rotator. Nuclei were precipitated by centrifugation (2,000 rcf, for 10 min at 48°C), and RNA was extracted from the supernatant using a standard RNeasy Mini Kit (Qiagen).

### BaseScope staining

A Cas9-transgenic THP-1 WT clone was nucleofected with GFP gRNA and sorted for GFP-negative cells. Cells were nucleofected as indicated in CRISPR-targeting of cell lines, with nontarget control gRNA, ADAR gRNA, and/or hnRNPC gRNA and grown for 4 d. Cells were centrifuged for 5 min at 350 rcf and RT, media were removed, and cells were resuspended in PBS. Cells were centrifuged again, PBS was carefully removed, and cells were resuspended in RNase-free 10% Neutral Buffered Formalin and incubated for 30 min at 37°C. Cells were precipitated again and resuspended in RNase-free, ice-cold 70% ethanol to obtain a cell concentration of 1e6/ml and stored at 4°C until used (max 1 mo). Samples were processed with Shandon Cytofunnel (Thermo Fisher Scientific; Cat. No. 1102548) and centrifuged at 800 rcf for 10 min, and the slides were removed from the cytoprep kit. Slides were air-dried for 20 min at RT and dehydrated in 50%, 70%, and 100% ethanol in preparation for staining.

#### Automated procedures

Automated cytospin BaseScope ISH is a modified single BaseScope ISH protocol from Advanced Cell Diagnostics (ACD) BaseScope LS Detection Protocol User Manual (323600 USM), performed using a Leica Bond-RX system. Pretreatment steps were adjusted to maintain an optimal sensitivity versus morphology for cytospin samples. The developed fluorescent ISH procedure was modified from ACD protocol in the amplification and detection steps. Samples were tested for RNA quality with a BA-Hs-PPIB probe before running with target probes, and bacteria gene DapB probe was used as a negative probe for procedure control.

#### Sample pretreatment

After cytospin, slides were removed from 100% EtOH and dried for 30 min in an oven at 37°C. Slides were then labeled with modified *ACD2.5 Red RevB as staining protocol (without Amp7, Amp8, and counterstaining steps) and inserted into the Bond RX slide rack trays to be processed. The “frozen slide delay” was selected as preparation protocol to accommodate the overnight delay run. Antigen retrieval was conducted with *ACD HIER 15 min with ER2 at 88°C, followed by the peroxide quenching step in the same protocol. HIER with Protease step was omitted to avoid overdigestion of the sample, which could lead to a nonspecific background in DapB-negative control probe.

#### Fluorescent BaseScope ISH procedure

The ACD 1-min hybridization protocol for hybridization step (ACD; 323600 USM) was selected. Fluorescent cytospin ISH procedure is a modified staining protocol of the single chromogenic BaseScope LS reagent kit (Cat. No. 323600). Following sample pretreatment, hybridization and amplification steps were done according to the ACD BaseScope LS Detection Reagent User Manual (323600 USM) from Amp 1 to Amp 6 steps. Probes were hybridized for 2 h at 42°C. Slides were washed with 1X Bond wash buffer (Leica 10X concentrate; AR9590) at 42°C three times (0, 1, and 5 min) followed by eight washes with 1X Bond wash buffer, 0 min each. Samples were processed only to the end of the Amplification 6 step (*ACD Amp 6) followed by washes. ISH detection was completed using Opal-570 (PerkinElmer; Cat. No. FP1488001KT; (1:1,500) in 1X amplification buffer (PerkinElmer; NEL794001KT) 1 and 10 min each at RT. Slides were washed with 1X Bond wash solution three times, 0 min each, followed by additional five times, 1 min each, at RT. Slides were counterstained with Spectral DAPI (PerkinElmer; FP1490), and counterstain was performed for 5 min at RT. Excess DAPI was rinsed off by five washes with deionized water. Finally, the slides were coverslipped with Prolong Gold antifade reagent (Life Technology; Cat No. P36930). BaseScope probes are listed in Table S5.

### Imaging

Single optical sections were acquired by sequential scanning on a LEICA Sp8 confocal microscope (controlled by LAS X version 3.55 software), using an HC PL APO CS2 40×/1.30 oil objective, with 1.5 zoom. The images are 1,024 × 1,024 pixels (pixel size, 0.189 × 0.189 μm), 194 × 194 μm physical size. The pinhole size was set to 1 Airy Unit (65.3 µm), resulting in 1.22-µm-thick optical sections.

To detect DAPI signal, a 405-nm diode laser was used with 20% output. The photomultiplier tube detector settings were 430–480-nm detector window with the gain set to 550 and the offset to −1. To detect the OPAL-570 signal, a 552-nm diode laser was used with 15% output. The photomultiplier tube settings were 570–620-nm detector window, with the gain set to 800 and the offset to −5.

Lookup tables were set to blue for DAPI and red for OPAL-570 dye.

All images were acquired under identical settings on the same day. The images shown are the original captures exported as TIFF files; no postacquisition modifications of any kind were applied to them.

### RNA-seq and alignment

The concentration of RNA was determined using NanoDrop 8000 (Thermo Fisher Scientific), and the integrity of RNA was assessed by Bioanalyzer 2100 using the Analyzer Eukaryote Total RNA Nano Chip (Agilent). Libraries were prepared using the TruSeq Stranded Total RNA Library Prep Kit with Ribo-Zero Gold kit (Illumina). The libraries were multiplexed and sequenced on Illumina HiSeq4000 (Illumina). An average of >130 million paired-end 100–bp reads for RNA-seq study 1 and >85 million paired-end 150-bp reads for RNA-seq study 2 was obtained per sample. Reads were aligned to GrCH37 v13 using STAR (V2.5.4a) with the basic two-pass mode allowing for a maximum number of multiple alignments of 1,000 and 999 mismatches per read (–outFilterMultimapNmax 1000–outFilterMismatchNmax 999) but 10% mismatches per read pair using –outFilterMismatchNoverReadLmax 0.1 to increase the detection of A-to-I editing sites. In addition, we allowed introns to be 20–1,000,000 nt and a 1,000,000-nt distance between read pair mates. BAM files were sorted using samtools sort, and a bai index was generated using samtools index.

### LeafCutter splicing analysis and mapping to RepeatMasker elements

The LeafCutter pipeline (version 0.2.7) for differential splicing was applied according to the Differential Splicing documentation hnRNPC-targeted versus WT samples, using the unsorted BAM files of all nine samples per treatment, independent of the incubation time (3, 4, or 5 d) as an input. To estimate the involvement of Alu splice sites to differential splicing, we mapped all intron ends as indicators of splice sites to GrCH37/hg19 RepeatMasker annotations using R GenomicRanges::mapToTranscripts. Splicing clusters were then stratified based upon the mapping of at least one splice site to an Alu element (Alu), no splice site mapping to an Alu but any splice site mapping to any other RepeatMasker element (non-Alu RE), or no splice site mapping to any RepeatMasker element (no RE).

### Differential relative use of splice sites and iClip overlap with splice sites

After significantly regulated splicing clusters (adjusted P value ≤ 0.001) were selected, intronends as equivalent to splice sites were quantified using the “_perind_numers.counts” output of the LeafCutter analysis. Intron counts were aggregated by genomic intron starts and ends separately, as an indicator for splice site use. Then, the relative use of splice sites was determined per splicing cluster by normalizing the count of each intronend (both intron starts and ends) to the total counts of each cluster (for illustration, see Fig. S3 C). Processed iCLIP clusters from Zarnack et al. (2013) were downloaded from the ArrayExpress accession no. E-MTAB-1371, and hnRNPC_iCLIP_all_clusters.bed was used. The bed file was imported to R as GRanges object, and a distance of maximum 50 nt of intronends to iCLIP clusters was determined using GRanges::findOverlaps with the option maxgap = 50.

### Differential expression analysis

Aligned reads were summarized to gene level using the R Rsubread package featureCounts function in paired end mode (isPairedEnd = TRUE) based on the GrCH37 gencode V19 GTF annotation, using the default exons as features for counting and genes as meta-features (GTF.featureType=“exon” and GTF.attrType=“gene_id”). We only allowed uniquely mapping reads by setting the read-quality filter to 255 (minMQS = 255).

To quantify introns and ECRs, we first extracted intron ranges from APPRIS annotation based on https://davetang.org/muse/2013/01/18/defining-genomic-regions/ from the GrCH37 gencode V19 GTF file using awk to filter for APPRIS-annotated transcripts (awk ’BEGIN’{OFS=“\t”;} /appris/ {print}’). Exons were extracted from the APPRIS-annotated transcripts into bed format using awk ('BEGIN{OFS=“\t”;} $3==”exon” {print $1,$4-1,$5,$10,$3}') and sorted using bedtools2 sortBed, and overlapping exons were merged with bedtools2 mergeBed. Next, unique genes of APPRIS-annotated transcripts were extracted using awk as described above, and their annotations were extracted from the original GTF file and sorted, and the merged APPRIS-annotated exons were subtracted from genes using bedtools2 subtractBed to yield introns delineated from APPRIS-annotated transcripts, saved in bed format for later quantification.

To define ECRs, A-to-I editing clusters were defined as described in Detection of A-to-I editing. For quantification, ECRs were quantified rather than editing clusters, to avoid quantification errors due to multimapping reads. Therefore, 1,000 bp were added to each cluster on both the 3′ and 5′ UTR ends, and these regions were merged using bedtools2 mergeBed, requiring at least 1-bp overlap. Reads mapping to intron and ECR features were counted using the R Rsubread package featureCounts function after converting bed files to SAF format, as described for gene quantification, not using the meta-feature flag. Low expressed genes were filtered before voom/limma differential expression analysis. For study 1, pairwise comparisons, all samples were included into the design matrix, treating each triplicate of samples as independent conditions. Gene, intron, and ECR counts were normalized to total genecounts corrected by edgeR normalization factors (calcNormFactors in default modus). The average log cpm filter cutoffs (across all samples) were 0.5 for gene counts, 1 for intron counts, and 4.5 for ECRs. Counts were transformed using the standard voom function and fit to a linear model using vmfit. Pairwise contrasts were fit with eBayes and differential expression analysis tables created using topTable with standard FDR-adjusted P value applied. Features were considered meaningfully regulated if they showed an absolute positive log2 (fold change) ≥1 and an FDR-corrected P value ≤0.05, if not specified otherwise.

For the generation of heatmaps and volcano plots, introns were checked for their overlap with RepeatMasker elements and assigned to genes applying bedtools intersect with flags -loj -a on bed files. Overlaps with RepeatMasker elements were prioritized in the following way: If an intron was overlapping with at least one Alu element, irrespective of other RepeatMasker elements, it was designated overlapping with an Alu; if any other RepeatMasker element but no Alu element overlapped, it was designated overlapping with a non-Alu RE. To estimate whether an intron overlapped with a differentially regulated splicing cluster, the most 3′ and 5′ UTR end of any intron within a splicing cluster was extracted from the LeafCutter_ds_effect_sizes.TXT file to define the region the cluster was spanning and the associated adjusted P value from the LeafCutter_ds_cluster_significance.TXT file, both generated by the LeafCutter pipeline. Then, introns and splicing cluster overlaps were discovered using R GenomicRanges::mapToTranscripts.

### Factorial design

Interaction between factor 1 (CTRL, hnRNPC) and factor 2 (CTRL, ADAR) in study 1 was estimated by 2 × 2 factorial design as described in the limma users’ guide as classic interaction model. For this, samples from day 5 were selected that were nucleofected with 2 × CTRL, CTRL+hnRNPC gRNA (hnRNPC single deficiency), CTRL + ADAR gRNA (ADAR single deficiency), or hnRNPC+ADAR gRNA (ADAR and hnRNPC combined deficiency). Genes induced by IFN-α (log2[fold change] ≥1 and FDR-adjusted P value ≤ 0.05) were selected, counts were normalized using voomWithQualityWeights using the original library sizes, and lmfit and eBayes were applied and topTable with option coef = 4 was used to extract FDR-adjusted P values for interaction between factor 1 and factor 2.

### Single-nucleotide polymorphism (SNP) detection

To be able to exclude SNPs as false-positive A-to-I editing sites, we subjected the STING-deficient Cas9 transgenic THP-1 clone to whole-genome sequencing: DNA was isolated using the Gentra Puregene Blood Kit (Qiagen). The concentration of DNA was determined using NanoDrop 8000 (Thermo Fisher Scientific). Library was prepared using the TruSeq DNA Nano Kit (Illumina) and sequenced on Illumina HiSeq2500 to generate ∼200 million paired-end 75-bp reads. Reads were trimmed using trimmomatic in paired-end mode with flags -phred33 ILLUMINACLIP:TruSeq3-SE.fa:2:30:10 LEADING:3 TRAILING:3 SLIDINGWINDOW:4:15 MINLEN:36. Paired reads were then aligned to hg19 using bwa mem with flag -M, and the output SAM file was converted to BAM format using samtools view, the BAM file was sorted using samtools sort, PCR duplicates were marked using Picard tools, an index was generated using samtools index, a sequence dictionary was generated using Picard tools, and variants were detected using GATK UnifiedGenotyper. Then, variants were filtered for SNPs using bcftools view with flags -v snps -Oz, and bedops convert2bed was used to convert the vcf output to a bed file, which was cut to bed3 as a filtering input for the SAILOR pipeline in A-to-I editing analysis.

### A-to-I editing analysis

To discover A-to-I editing sites, we used the SAILOR pipeline (version 1.0.4) described before (Deffit et al., 2017), using the default settings except reverse_stranded_library: true, single_end: false and min_variant_coverage: 10. We used the bed3 file generated in SNP detection to exclude genomic SNPs. Following discovery, editing sites were subjected to several filtering steps. First, each site was required to have all of the following qualities in at least one ADAR WT sample: passing of the SAILOR-set filter, a minimum SAILOR-score of 0.5, minimum coverage of 10 (any nucleotide), minimum editing frequency of 0.05, and maximum editing frequency of 0.95. Editing sites were assigned to clusters using the clusterMaker function of the R bumphunter package, with a maximum distance of 50 between sites within one cluster (maxGap = 50). Since editing sites missing in the final per-sample SAILOR output could be filtered due to low expression or lack of editing, base-specific counts per editing site were extracted again from SAILOR-generated BAM files sorted by strand, filtered for PCR duplicates and for other filters employed by the SAILOR pipeline (*.sorted.rmdup.readfiltered.bam). These were fed into samtools mpileup with flags -B -d10000000 -t DPR,INFO/DPR,DP4 -uf, using the -l flag with a bed annotation of the primary selection of editing sites. INFO/DPR counts were used to calculate the editing frequency. T and C counts for sense or A and G counts for antisense reads, respectively, were considered sequencing errors and were added to reference counts. Sites were filtered once more. First, not available values for read counts and frequency were set to 0. Then, for a site to be retained, the following was required: all samples ≥0.05 editing frequency, two samples ≥10% editing frequency, all samples ≥10 read counts. This was required for the three samples of at least one treatment. Finally, editing sites were clustered again, and only sites that were part of a cluster with at least five sites were retained as the final selection of editing sites.

For the mapping of editing sites to repetitive elements, University of California, Santa Cruz GrCH37/hg19 RepeatMasker annotations were used and mapped using bedtools intersect with the -loj flag. If a site mapped to Alu, irrespective of its mapping to another RepeatMasker element, it was considered mapping to Alu. If it was mapping to any other RepeatMasker element, it was labeled mapping to a non-Alu RE (RepeatMasker element). If it was not mapping to any RepeatMasker element (output “.”), it was labeled non-RE. For plotting, editing sites with ≥10 read counts for all samples in a comparison were selected. Average editing per site per treatment was calculated as sum(edit counts)/sum(read counts) across all samples of each treatment, irrespective of treatment time (days 3, 4, and 5). Average editing per ECR was calculated as sum(edit counts)/sum(read counts) across all editing sites and all three samples of each treatment, in a day-wise manner. Only high-quality mpileup INFO/DPR edit and read counts (see SAMtools documentation) were used.

To enable mapping of editing clusters to genic elements, 3′ UTR, 5′ UTR, coding sequence (CDS), exon, intron, and whole-gene tracks were downloaded from the University of California, Santa Cruz table browser using the GrCH37/hg19 assembly, Gencode Genes V19 tracks, and the Basic table. Editing clusters were then mapped to genomic regions using bedtools intersect with -loj -a flags. In case of mapping to multiple features, mapping was prioritized as follows: CDS > 3′ UTR > 5′ UTR > exon > intron > gene > no intersect (“intergenic”). To determine the distance between editing clusters and upstream 3′ UTRs, bed files were imported to R as GenomicRanges, and the nearest upstream 3′ UTR of each intergenic editing cluster was retrieved by using GenomicGanges::follow in a strand-dependent manner and determining the distance between the UTRs and editing clusters with GenomicRanges::distance.

### Characterization of secondary structures

Sequences for L1, preLet7b, hAT2, and ES27L hairpins were downloaded from Ahmad et al. (2018), and NICN1, BPNT1, DESI1 irAlu, and ECR sequences were extracted from hg19 using samtools faidx. From sequences of RNAs encoded on the (−)−strand, reverse complement of the sequence was generated. Sequences as DNA sequences were input into viennaRNA v.2.4.13 RNAfold, and secondary structures were plotted using viennaRNA v.2.4.13 RNAplot. Long dsRNA stretches from controls or dsRNA stretches from ECRs >250 nt, as well as the longest dsRNA stretch within the structure, were manually determined from the plot. The respective dot-bracket structure annotation was extracted from the RNAfold output. (number of “.” within dot-bracket structure)/(length of dot-bracket structure) determined the fraction of mismatched nucleotides.

### Online supplemental material

Fig. S1 contains eCLIP AluRNA enrichment as well as supporting material for Fig. 1 B (IFIT1/IFIT2 expression) and Fig. 1 C (Western immunoblot). Fig. S2 shows ISG expression in hnRNPC- and ADAR-deficient cells in further cell lines as well as primary monocytes. Fig. S3 illustrates differential splicing analysis, intron expression analysis, and examples of intergenic editing clusters. Fig. S4 shows further plots comparing localization and dysregulation of ECRs upon hnRNPC deficiency as well as full micrographs of BaseScope analysis in Fig. 9 C. Fig. S5 provides a schematic illustration of the proposed model. Table S1 shows references for Fig. S1, A and B. Table S2 contains crRNA sequences. Table S3 lists the exact gRNAs used for each figure. Table S4 details SYBR green primers, and Table S5 shows BaseScope probes used in this study. Supplementary data contain tables including column descriptions used to plot RNA-seq–based figures: Data S1, Fig. 2 B (differential gene expression); Data S2, Fig. 2 C (ISG expression); Data S3, Fig. 4 B (differential intron expression); Data S4, Fig. 5, A, C, and E (normalized expression of introns and respective genes); Data S5, Fig. 7, B and C (differential ECR expression); Data S6, Fig. 7 E (average editing in ECRs); Data S7, Fig. 9 A (differential gene expression cytosol versus total RNA); and Data S8, Fig. 9 B and Fig. S4 A (ECR expression cytosol versus total RNA and ADAR versus ADAR+hnRNPC–deficient cells).

## Supplementary Material

Table S1 lists references for Fig. S1.

Table S2 lists crRNA sequences.

Table S3 lists gRNAs used, by figure.

Table S4 lists SYBR green qPCR primer sequences.

Table S5 lists BaseScope probes.

Data S1 lists differential gene expression analyses.

Data S2 presents ISG expression.

Data S3 lists differential intron expression analyses.

Data S4 shows normalized expression of introns and respective genes.

Data S5 lists differential ECR expression analyses.

Data S6 shows average editing in ECRs.

Data S7 lists differential gene expression analysis, total versus cytosolic RNA.

Data S8 shows data for differential ECR expression versus differential ECR localization.

## Data Availability

Sequencing data and raw counts are available under Gene Expression Omnibus accession number GSE176012. Results from analyses supporting the findings of this study are available within the paper and its supplementary information files. Code is available through the corresponding author A.-M. Herzner upon request.
