# Supplementary material for: ADAR and hnRNPC deficiency synergize in activating endogenous dsRNA-induced type I IFN responses
Source: J Exp Med. 2021 Jul 23;218(9):e20201833. doi: 10.1084/jem.20201833 (PMC8313407; doi:10.1084/jem.20201833)
Supplement: Table S1 — lists references for Fig. S1. [file JEM_20201833_TableS1.docx]

Table S1. References for Fig. S1

| Protein | Interacting with Alu-RNA | Alu-RNA related function | Role in auto-immune disease | Antibodies in autoimmunity |
| --- | --- | --- | --- | --- |
| hnRNPA1 | Donev et al., 2003; Pastor and Pagani, 2011; Donev et al., 2007 | Donev et al., 2003; Pastor and Pagani, 2011; Howard et al., 2018 |  | Astaldi Ricotti et al., 1989; Lee et al., 2006; Vordenbäumen et al., 2016; Levin et al., 2017 |
| hnRNPC | Zarnack et al., 2013 | Zarnack et al., 2013; Tajnik et al., 2015; Attig et al., 2016; Jourdy et al., 2018 |  | Vencovský et al., 1997; Heegaard et al., 2000 |
| ILF3 | Li et al., 2017 | (Li et al., 2017; Quinones-Valdez et al., 2019; Freund et al., 2020 |  |  |
| NONO | Chen et al., 2008 | Chen et al., 2008 |  | Vordenbäumen et al., 2016 |
| SRSF1 |  |  | Moulton et al., 2014; Katsuyama et al., 2019 |  |
| TIA1/TIAL1 | Gal-Mark et al., 2009 | Gal-Mark et al., 2009 |  | Jimenez-Boj et al., 2008 |
