# Supplementary material for: ADAR and hnRNPC deficiency synergize in activating endogenous dsRNA-induced type I IFN responses
Source: J Exp Med. 2021 Jul 23;218(9):e20201833. doi: 10.1084/jem.20201833 (PMC8313407; doi:10.1084/jem.20201833)
Supplement: Table S2 — lists crRNA sequences. [file JEM_20201833_TableS2.docx]

Table S2. crRNA sequences

| crRNA name | crRNA variable sequence (5′ → 3′) |
| --- | --- |
| NTC1a | CGTTAATCGCGTATAATACG |
| NTC2a | CATATTGCGCGTATAGTCGC |
| NTC3a | GGCGCGTATAGTCGCGCGTA |
| EWSR1.1 | TACACCGCCCAGCCCACTCA |
| EWSR1.2 | TGCATAGGCGGTCTGCCCAT |
| EWSR1.3 | TGCCCATAGGCTGGATAAGC |
| hnRNPA1.1 | TGGATGCAGCTATGAATGCA |
| hnRNPA1.2 | CTTTGACGACCATGACTCCG |
| hnRNPA1.3 | AATGACAACTTCGGTCGTGG |
| hnRNPC.1 | CTCATTAACATACTGAACGA |
| hnRNPC.2 | GAGATTCCCAATGAATACAC |
| hnRNPC.3 | GATCTGCAGCGGAGATGTAC |
| hnRNPU.1 | CATCTGGTCGCCGTCCAGAG |
| hnRNPU.2 | GTCGCCGTTCTCGTCTTCCG |
| hnRNPU.3 | TTTGCCTTTTGACACACCAT |
| ILF3.1 | AATGCTTTGCCATCACATGG |
| ILF3.2 | CACATGACCAGAACCCTGCG |
| ILF3.3 | GCCCATGAAACGCCCAATGG |
| ILF3.4 | CAGCTGGTTCAACCGCATCA |
| LIN28B.1 | CATCGACTGGAATATCCAAG |
| LIN28B.2 | CCGTATTGACTCAAGGCCTT |
| LIN28B.3 | ATACGGGTAACAGGACCTGG |
| NCBP2.1 | GCTGCGCAGCGACTCCTACG |
| NCBP2.2 | ATATTACTCACGCGCAGATG |
| NCBP2.3 | AGGGCAGGCAATACGGCCGT |
| NONO.1 | CTGGACAATATGCCACTCCG |
| NONO.2 | ATTTCCTCGTCCTGTGACTG |
| POLR2G.1 | AAGTTGGGGCCGAAGTAGCG |
| POLR2G.2 | GCAGAAGCTCTTCACCGAGG |
| POLR2G.3 | TTTCCGGCCATTTAAAGGGG |
| PPIL4.1 | TCGACTTGTACACCGAAGAA |
| PPIL4.2 | TACAAACTGGCGATCCTACA |
| PPIL4.3 | CAAAGATAGACTCTCCTCCA |
| PTBP1.1 | ATTGTCCCAGATATAGCCGT |
| PTBP1.2 | AAAGGTGACAGCCGAAGTGC |
| PTBP1.3 | GCTCCCCATCGACGTCACGG |
| QKI.1 | GGATCTTCAACCACCTCGAG |
| QKI.2 | ACAATAGGTCCCACAGCATC |
| QKI.3 | GGATGTAAAATCATGGTCCG |
| RBM27.1 | ACATAGTTGGCTAAGGCTGA |
| RBM27.2 | GGCTTTACTGGTTCCAAAAG |
| RBM27.3 | GATTCTGAACGAGTCTTCTG |
| RPLP0.1 | TGATGCGCAAGGCCATCCGA |
| RPLP0.2 | TTATCCGAAATGTTTCATTG |
| RPLP0.3 | AGATCCGCATGTCCCTTCGC |
| RPS2.1 | AACCGCGGTGGCTTCCGCGG |
| RPS2.2 | CTTGACCAAGCGGCCCAACT |
| RPS2.3 | GATCATTGATTTCTTCCTGG |
| SRSF1.1 | GCGGCAATCGTTGTTCCCTG |
| SRSF1.2 | ACCTCCAGACATCCGAACCA |
| SRSF1.3 | AACTCAACGAAGGCGAAGGG |
| SRSF9.1 | TCTCGCGCACGTCGGTCGGA |
| SRSF9.2 | ATCGAGCTCAAGAACCGGCA |
| SRSF9.3 | CCCGACCTCCATAAGTCCTG |
| STAU2.1 | TCTAATGGCCTGTAGATGGC |
| STAU2.2 | TTTTATGTTCAGCTCACTGT |
| STAU2.3 | GGTGGTCGCAGCGCGCTTCT |
| TIA1.1 | AGTTTTTACAAGGTCCAATC |
| TIA1.2 | CATTAGCTGCTATGAATGGA |
| TIA1.3 | TGAGATCACCAACAAAGACA |
| TIAL1.1 | TGCCCGGGTAGTTAAAGACA |
| TIAL1.2 | AGATACAAAACCATAGCCTT |
| TIAL1.3 | AATGCGATTGTGCATATGGG |
| ADAR.1 | TTCTTGTAGGGTGAACACCG |
| ADAR.2 | TATATCTCGGGCCTTGGTA |
| RIGIb | AGCCTTCCAGGATTATATC |
| MDA5b | TAGCGGAAATTCTCGTCTG |
| STING.1 | CAGGCACTCAGCAGAACCA |
| STING.2 | GCCACCAGAGCACACTCTC |
| SETDB1.6b | GTGGAAGTCCCGAGTTGAGG |
| CD81b | GTTGGCTTCCTGGGCTGCTA |
| GFP | AAGTTCAGCGTGTCCGGCGA |
| TREX1 | ACATCTGTGGACAGCCAGC |
| UPF1 | CTGCTTGGCGCCGACACACA |

^a^IDT controls.

^b^Reference: Cuellar et al., (2017).
