# Supplementary material for: ADAR and hnRNPC deficiency synergize in activating endogenous dsRNA-induced type I IFN responses
Source: J Exp Med. 2021 Jul 23;218(9):e20201833. doi: 10.1084/jem.20201833 (PMC8313407; doi:10.1084/jem.20201833)
Supplement: Table S3 — lists gRNAs used, by figure. [file JEM_20201833_TableS3.docx]

Table S3. gRNAs used, by figure

| Position/label | gRNA #1 | gRNA #2 | gRNA #3/Treatment |
| --- | --- | --- | --- |
| **Fig. 1 B and Fig. S1, C and D: Cell line, STING-deficient cas9-transgenic THP-1** | | | |
| 1 | Buffer only | Buffer only | – |
| 2 | NTC1 | NTC1 | – |
| 3 | ADAR.1 | NTC1 | – |
| 4 | ADAR.1 | NTC2 | – |
| 5 | ADAR.1 | NTC3 | – |
| 6 | ADAR.1 | CD81 | – |
| 7 | ADAR.1 | GFP | – |
| 8 | ADAR.1 | TREX1 | – |
| 9 | ADAR.1 | SETDB1 | – |
| 10 | ADAR.1 | ESWR1.1 | – |
| 11 | ADAR.1 | ESWR1.2 | – |
| 12 | ADAR.1 | ESWR1.3 | – |
| 13 | ADAR.1 | hnRNPA1.1 | – |
| 14 | ADAR.1 | hnRNPA1.2 | – |
| 15 | ADAR.1 | hnRNPA1.3 | – |
| 16 | ADAR.1 | hnRNPC.1 | – |
| 17 | ADAR.1 | hnRNPC.2 | – |
| 18 | ADAR.1 | hnRNPC.3 | – |
| 19 | ADAR.1 | hnRNPU.1 | – |
| 20 | ADAR.1 | hnRNPU.2 | – |
| 21 | ADAR.1 | hnRNPU.3 | – |
| 22 | ADAR.1 | ILF3.1 | – |
| 23 | ADAR.1 | ILF3.2 | – |
| 24 | ADAR.1 | ILF3.3 | – |
| 25 | ADAR.1 | ILF3.4 | – |
| 26 | ADAR.1 | LIN28B.1 | – |
| 27 | ADAR.1 | LIN28B.2 | – |
| 28 | ADAR.1 | LIN28B.3 | – |
| 29 | ADAR.1 | NCBP2.1 | – |
| 30 | ADAR.1 | NCBP2.2 | – |
| 31 | ADAR.1 | NCBP2.3 | – |
| 32 | ADAR.1 | NONO.1 | – |
| 33 | ADAR.1 | NONO.2 | – |
| 34 | ADAR.1 | POLR2G.1 | – |
| 35 | ADAR.1 | POLR2G.2 | – |
| 36 | ADAR.1 | POLR2G.3 | – |
| 37 | ADAR.1 | PPIL4.1 | – |
| 38 | ADAR.1 | PPIL4.2 | – |
| 39 | ADAR.1 | PPIL4.3 | – |
| 40 | ADAR.1 | PTBP1.1 | – |
| 41 | ADAR.1 | PTBP1.2 | – |
| 42 | ADAR.1 | PTBP1.3 | – |
| 43 | ADAR.1 | QKI.1 | – |
| 44 | ADAR.1 | QKI.2 | – |
| 45 | ADAR.1 | QKI.3 | – |
| 46 | ADAR.1 | SRSF1.1 | – |
| 47 | ADAR.1 | SRSF1.2 | – |
| 48 | ADAR.1 | SRSF1.3 | – |
| 49 | ADAR.1 | SRSF9.1 | – |
| 50 | ADAR.1 | SRSF9.2 | – |
| 51 | ADAR.1 | SRSF9.3 | – |
| 52 | ADAR.1 | STAU2.1 | – |
| 53 | ADAR.1 | STAU2.2 | – |
| 54 | ADAR.1 | STAU2.3 | – |
| 55 | ADAR.1 | TIA1.1 | – |
| 56 | ADAR.1 | TIA1.2 | – |
| 57 | ADAR.1 | TIA1.3 | – |
| 58 | ADAR.1 | TIAL1.1 | – |
| 59 | ADAR.1 | TIAL1.2 | – |
| 60 | ADAR.1 | TIAL1.3 | – |
| **Fig. 1, C and F (right): STING-deficient cas9-transgenic THP-1** | | | |
| 1 | NTC1 | NTC1 | – |
| 2 | ADAR.2 | NTC1 | – |
| 3 | NTC1 | hnRNPC.2 | – |
| 4 | ADAR.2 | hnRNPC.2 | – |
| **Fig. 1 D: STING-deficient cas9-transgenic THP-1** | | | |
| 1 | NTC1 | NTC1 | NTC1 |
| 2 | NTC1 | NTC1 | RIGI |
| 3 | NTC1 | NTC1 | MDA5 |
| 4 | NTC1 | hnRNPC.1 | NTC1 |
| 5 | NTC1 | hnRNPC.1 | RIGI |
| 6 | NTC1 | hnRNPC.1 | MDA5 |
| 7 | ADAR.1 | NTC1 | NTC1 |
| 8 | ADAR.1 | NTC1 | RIGI |
| 9 | ADAR.1 | NTC1 | MDA5 |
| 10 | ADAR.1 | hnRNPC.1 | NTC1 |
| 11 | ADAR.1 | hnRNPC.1 | RIGI |
| 12 | ADAR.1 | hnRNPC.1 | MDA5 |
| **Fig. 1 F (left): STING-deficient cas9-transgenic THP-1** | | | |
| 1 | NTC1 | – | Opti-MEM |
| 2 | NTC1 | – | pI:C |
| 3 | hnRNPC.2 | – | Opti-MEM |
| 4 | hnRNPC.2 | – | pI:C |
| **Fig. 1 E: STING-deficient cas9-transgenic THP-1** | | | |
| 1 | NTC1 | NTC1 | NTC1 |
| 2 | NTC1 | hnRNPC.2 | NTC1 |
| 3 | ADAR.2 | NTC1 | NTC1 |
| 4 | NTC1 | NTC1 | RIGI |
| 5 | NTC1 | NTC1 | MDA5 |
| **Fig. 2; Fig. 3, A, B, and E; Fig. 4; Fig. 5; Fig. 6; Fig.7; Fig. S1, A and B; Fig. S3; Fig. S4 A: STING-deficient cas9-transgenic THP-1** | | | |
| CTRL/NTC/non-target control | NTC1 | NTC1 | Medium |
| hnRNPC (single) | NTC1 | hnRNPC.2 | Medium |
| ADAR (single) | ADAR.2 | NTC1 | Medium |
| ADAR+hnRNPC double | ADAR.2 | hnRNPC.2 | Medium |
| GFP | NTC1 | GFP | Medium |
| IFN-α treatment^a^ | NTC1 | NTC1 | IFN-α 1,000 U/ml |
|  | | | |
| **Fig. 3 C: STING-deficient cas9-transgenic THP-1** | | | |
| 1 | NTC1 | NTC1 | – |
| 2 | ADAR.1 | NTC1 | – |
| 3 | NTC1 | hnRNPC.1 | – |
| 4 | ADAR.1 | hnRNPC.1 | – |
| **Fig. 3 D, Fig. S2 E: MCF-7** | | | |
| 1 | NTC1 | NTC1 | – |
| 2 | ADAR.2 | NTC1 | – |
| 3 | NTC1 | hnRNPC.2 | – |
| 4 | ADAR.2 | hnRNPC.2 | – |
| **Fig. 3, F and G: STING-deficient cas9-transgenic THP-1** | | | |
| 1 | NTC1 | NTC1 | NTC1 |
| 2 | NTC1 | NTC1 | UPF1 |
| **Fig. 3 H, Fig. 8** | | | |
| 1 | NTC1 | NTC1 | NTC1 |
| 2 | NTC1 | NTC1 | UPF1 |
| 3 | ADAR.1 | NTC1 | NTC1 |
| 4 | ADAR.1 | NTC1 | UPF1 |
| 5 | NTC1 | hnRNPC.1 | NTC1 |
| 6 | NTC1 | hnRNPC.1 | UPF1 |
| 7 | ADAR.1 | hnRNPC.1 | NTC1 |
| 8 | ADAR.1 | hnRNPC.1 | UPF1 |
| **Fig. 9, A, B, and D; Fig. 10; Fig. S4 A: WT cas9-transgenic THP-1 clone** | | | |
| CTRL/NTC/nontarget control | NTC1 | NTC1 | – |
| hnRNPC (single) | NTC1 | hnRNPC.1 | – |
| ADAR (single) | ADAR.1 | NTC1 | – |
| ADAR+hnRNPC double | ADAR.1 | hnRNPC.1 | – |
| **Fig. 9 C, Fig. S4 B: GFP-deficient, cas9-transgenic THP-1 clone** | | | |
| 1 | NTC1 | NTC1 | – |
| 2 | ADAR.2 | NTC1 | – |
| 3 | NTC1 | hnRNPC.2 | – |
| 4 | ADAR.2 | hnRNPC.2 | – |
| **Fig. S1 E: STING-deficient cas9-transgenic THP-1** | | | |
| 1^b^ | NTC1 | NTC1 | – |
| 2^b^ | NTC1 | hnRNPC.2 | – |
| 3 | NTC2 | hnRNPC.3 | – |
| 4^b^ | ADAR.2 | NTC1 | – |
| 5^b^ | ADAR.2 | hnRNPC.2 | – |
| 6 | ADAR.2 | hnRNPC.3 | – |
|  | |  |  |
| **Fig. S2, A–C: Parental cas9 transgenic cell line, WT clone, alternative STING-deficient clone** | | | |
| 1 | NTC1 | NTC1 | – |
| 2 | ADAR.1 | NTC1 | – |
| 3 | NTC | hnRNPC.1 | – |
| 4 | ADAR.1 | hnRNPC.1 | – |
| **Fig. S2 D: Primary monocytes/macrophages** | | | |
| 1 | NTC1 | NTC1 | – |
| 2 | NTC1 | hnRNPC.2 | – |
| 3 | ADAR.2 | NTC | – |
| 4 | ADAR.2 | hnRNPC.2 | – |

^a^IFN-α was added on day 3; cells were harvested 24 h later.

^b^Used in Fig. 1 C.
