# Supplementary material for: ADAR and hnRNPC deficiency synergize in activating endogenous dsRNA-induced type I IFN responses
Source: J Exp Med. 2021 Jul 23;218(9):e20201833. doi: 10.1084/jem.20201833 (PMC8313407; doi:10.1084/jem.20201833)
Supplement: Table S4 — lists SYBR green qPCR primer sequences. [file JEM_20201833_TableS4.docx]

Table S4. SYBR green qPCR primer sequences

| Assay | Primer 1 (5′ → 3′) | Primer 2 (5′ → 3′) |
| --- | --- | --- |
| CASC3 | CATGATCTTCGAGGGCAAAC | TCCATAGCTTTCGCTGACG |
| SMG5^a^ | ACCTCCCCTCTAGCCTTCTG | CGCACCACTGACTCCTCTAA |
| BRD8 splice 1^b^ | CATGGTCCATCCGAGAGAAG | GTGACAGTGAGACCCTGTCAA |
| BRD8 splice 2^b^ | GTTCTGCAAAGGGCTTGATT | ACCACAGGCCGCAGGGTA |
| RCN3 splice^b^ | GGGAACTTCCAGTACGACCA | GTCACTGCACTCCAGCCC |
| ZNF367 splice^b^ | CTGCTCCTGATCAGCCTTCT | CTGGAGTAGCTGGGATTACAGG |
| OSTC splice^b^ | TTCATTGACAGGAGCAAATCC | TGCACTACCACACCCAGGGG |

^a^Reference: Attig et al. (2016).

^b^Primers 2 span intron between Alu-exon and regular exon. Bold, underlined are nucleotides matching regular exon.
