# Supplementary material for: ADAR and hnRNPC deficiency synergize in activating endogenous dsRNA-induced type I IFN responses
Source: J Exp Med. 2021 Jul 23;218(9):e20201833. doi: 10.1084/jem.20201833 (PMC8313407; doi:10.1084/jem.20201833)
Supplement: Table S5 — lists BaseScope probes. [file JEM_20201833_TableS5.docx]

Table S5. BaseScope probes

| Probe names | Vendor | Catalog number |
| --- | --- | --- |
| BaseScope 2.5 LS Human-BRD8-O1-3zz-st | ACD Biosystems | 883738 |
| BaseScope 2.5 LS Human-TMEM116-O1-3zz-st | ACD Biosystems | 884288 |
| BaseScope 2.5 LS Human-MIGA1-O1-3zz-st | ACD Biosystems | 883728 |
| BaseScope 2.5 LS Human-PPIB | ACD Biosystems | 701038 |
| BaseScope 2.5 LS DapB | ACD Biosystems | 701018 |
